# Potent Cytotoxic Peptides from the Australian Marine Sponge *Pipestela candelabra*

**DOI:** 10.3390/md12063399

**Published:** 2014-06-04

**Authors:** Trong D. Tran, Ngoc B. Pham, Gregory A. Fechner, John N. A. Hooper, Ronald J. Quinn

**Affiliations:** Eskitis Institute for Drug Discovery, Griffith University, Brisbane, Queensland 4111, Australia; E-Mails: trong.tran@griffithuni.edu.au (T.D.T.); n.pham@griffith.edu.au (N.B.P.); gregfeq@gmail.com (G.A.F.); john.hooper@qm.qld.gov.au (J.N.A.H.)

**Keywords:** *Pipestela candelabra*, cytotoxicity, peptide, milnamide, hemiasterlin, geodiamolide, prostate cancer

## Abstract

Two consecutive prefractionated fractions of the Australian marine sponge extract, *Pipestela candelabra*, were identified to be selectively active on the human prostate cancer cells (PC3) compared to the human neonatal foreskin fibroblast non-cancer cells (NFF). Twelve secondary metabolites were isolated in which four compounds are new small peptides. Their structures were characterized by spectroscopic and chemical analysis. These compounds inhibited selectively the growth of prostate cancer cells with IC_50_ values in the picomolar to sub-micromolar range. Structure-activity relationship of these compounds is discussed.

## 1. Introduction

*Pipestela* has been characterized as a new sponge genus sharing some shape features and molecular DNA sequences with ten other genera in the family Axinellidae: *Auletta*, *Axinella*, *Cymbastela*, *Dragmacidon*, *Dragmaxia*, *Pararhaphoxya*, *Phakellia*, *Phycopsis*, *Ptilocaulis*, and *Reniochalina* [1]. The genus *Pipestela* is distributed only across the north-eastern region of Australia, and is very common from the Great Barrier Reef, Coral Sea, Papua New Guinea, Solomon Islands, and Vanuatu [2]. Chemical investigation of the sponge *P*. *candelabra* was first reported in 2012 with the identification of four cyclodepsipeptides, including jaspamide and pipestelides A–C, from a sample collected in the Solomon Islands [3]. However, a different structure class, hemiasterlin and milnamide analogues, was found in this chemical study on the Australian sponge *P*. *candelabra*.

Hemiasterlins and milnamides are two related small cytotoxic peptide families from marine sponges. Their structural features contain a dipeptide side chain of two unnatural amino acid residues, *tert*-leucine and *N*-methylvinylogous valine. This dipeptide fragment is incorporated with another atypical amino acid residue tri- or tetra-methylated tryptophan to produce the hemiasterlins or connected to a tetrahydro-β-carboline residue to form the milnamides. So far, six hemiasterlin derivatives (hemiasterlin or milnamide B [4], hemiasterlin A [5,6], hemiasterlin B [5], hemiasterlin C [6], crinamide A [5] and crinamide B [5]), and three milnamide analogues (milnamide A [7], milnamide C [8] and milnamide D [8,9]) have been identified from four sponge genera including *Auletta* [6,7], *Cymbastela* [5,9], *Hemiasterella* [4], and *Siphonochalina* [6]. These related natural products show potent cytotoxicity against many cancer cell lines, such as murine leukemia P388, human breast cancer MCF7, human glioblastoma/astrocytoma U373, human ovarian cancer HEY, human colon cancers LOVO and HT-29, human lung cancer A549, and murine melanoma B16-F10 [5,7]. In addition, hemiasterlin was a more potent *in vitro* cytotoxin and antimitotic agent than either of the anticancer drugs taxol or vincristine [10]. This made it stand out as an extremely exciting natural product lead structure for an anticancer drug development program. Many synthetic methodologies have been developed to produce a large number of hemiasterlin analogues [10,11,12,13]. From structure-activity relationship studies, the *tert*-leucine and *N*-methylvinylogous valine residues were found to be essential for bioactivity [10]. In 2003, the first hemiasterlin analogue HTI-286 (**13**) was selected for evaluation in a phase I clinical trial in patients with advanced solid tumors [10,14]. A phase II trial of HTI-286 has been halted since there were no objective responses and common toxicities observed included neutropaenia, nausea, alopecia, and pain [15]. However, there remains a particular interest to develop and examine the therapeutic effect of novel hemiasterlin derivatives. In 2009, another hemiasterlin analogue E7974 (**14**) with the *N*-isopropyl-d-pipecolic acid substituent replacing the *N*-methyltryptophan residue showed strong *in vivo* antitumor efficacy in many human xenograft cancer models and overcame a second mechanism of drug resistance present in cancer cells [16]. Results from phase I clinical trial have recently revealed that the compound E7974 remains a promising candidate for the treatment of several forms of advanced solid tumors in colorectal, pancreatic and liposarcoma cancer patients [17]. Human clinical testing of E7974 is currently ongoing [17].

**Figure 1:**
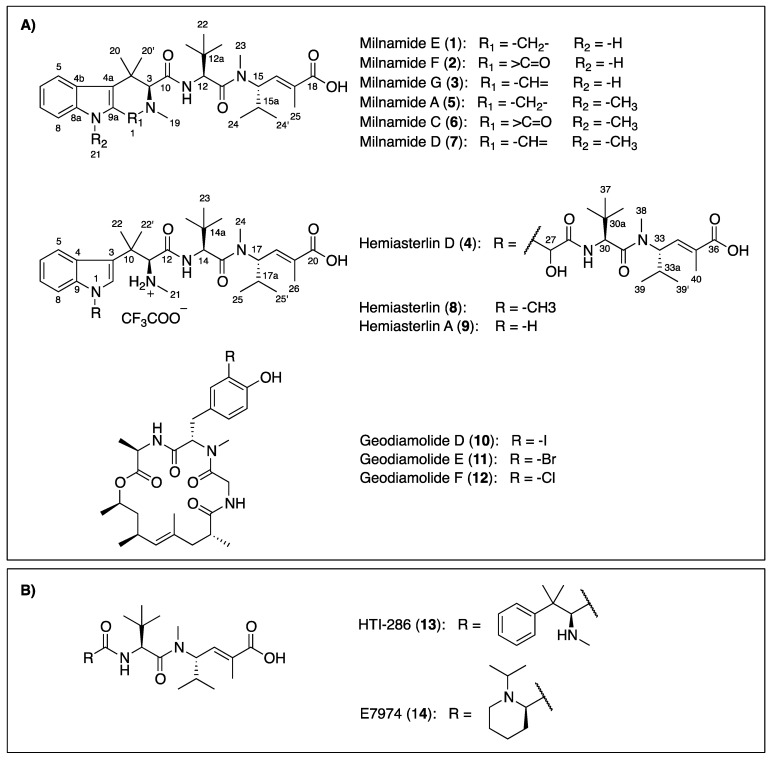
Chemical structures of compounds isolated from the Australian sponge *P*. *candelabra* (**A**) and related-hemiasterlin anticancer agents in clinical trial (**B**).

In the search for potential antitumor compounds from marine organisms, a subset of fractions in our Nature Bank prefractionated natural product library [18] was screened in cell-based cytotoxicity assays. Two consecutive fractions derived from the Australian sponge *Pipestela candelabra* were identified with a selective activity on the PC3 cancer cells compared to the NFF noncancer cells. This paper outlines the isolation, structure elucidation of four new compounds, milnamides E–G (**1**–**3**) and hemiasterlin D (**4**), along with eight known compounds including milnamide A (**5**) [7,9], milnamide C (**6**) [8], milnamide D (**7**) [8,9], hemiasterlin (or milnamide B) (**8**) [4,5,6,8,9], hemiasterlin A (**9**) [5,6], geodiamolide D (**10**) [19,20], geodiamolide E (**11**) [19,20], and geodiamolide F (**12**) [19,20] from two collections of the Australian sponge *P*. *candelabra* (Figure 1). Although hemiasterlins, milnamides and geodiamolides have been found co-occurring in *Hemiasterella minor* [4], *Cymbastela* sp. [5,9] and *Auletta* sp. [6,7], this is the first report of them from the sponge *Pipestela candelabra*.

## 2. Results and Discussion

**Figure 2:**
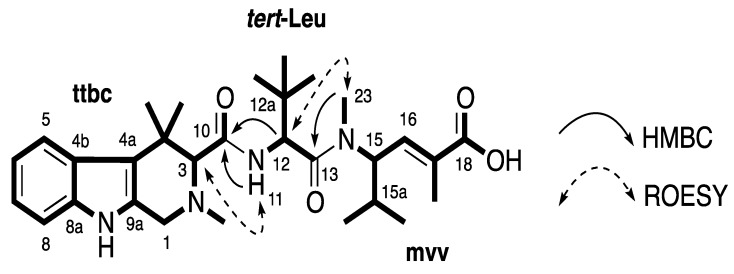
Partial structures (in bold) and key HMBC and ROESY correlations of **1**.

Compound **1** was obtained as a white amorphous solid. The (+)-HRESIMS displayed a pseudomolecular ion peak [M + H]^+^ ([C_30_H_45_N_4_O_4_]^+^) at *m/z* 525.3418, which was consistent with the molecular formula C_30_H_44_N_4_O_4_. Compound **1** was first isolated as a trifluoroacetic acid (TFA) salt and had broad signals in the ^1^H nuclear magnetic resonance (NMR) spectrum. Better NMR resolution was obtained after converting this compound from its TFA salt to formic acid (FA) salt. Combined data from ^13^C NMR and gHSQCAD spectra (Table 1) revealed three carbonyls (δ_C_ 171.0, 169.7, and 168.6 ppm), 10 aromatic and olefinic carbons (δ_C_ 138.2, 135.9, 131.8, 131.7, 125.4, 119.5, 118.6, 117.9, 113.0, and 110.9 ppm), two *N*-methyl carbons (δ_C_ 43.0 and 30.8 ppm), and 15 methine and methyl carbons (δ_C_ 72.1, 55.8, 53.4, 48.6, 34.7, 34.3, 30.0, 28.8, 3 × 26.4, 24.1, 19.3, 18.8, and 13.5 ppm). An indole ring system was deduced based on an ABCD spin system with signals at δ_H_ 7.47 (H-5, d, *J* = 8.4 Hz), 6.87 (H-6, t, *J* = 7.2, 7.8 Hz), 6.95 (H-7, t, *J* = 7.2, 7.8 Hz), and 7.24 (H-8, d, *J* = 8.4 Hz), an exchangeable proton NH (H-9) at δ_H_ 10.62 ppm and HMBC correlations from H-5 to C-4a (δ_C_ 113.0 ppm), from H-6 to C-4b (δ_C_ 125.4 ppm), from H-7 to C-8a (δ_C_ 135.9 ppm) and from the NH signal to C-4a, C-4b, C-8a and C-9a (δ_C_ 131.8 ppm). Further HMBC analysis indicated the correlations from H-1 (δ_H_ 3.62/3.88 ppm) to C-3 (δ_C_ 72.1 ppm), C-4a, C-9a and an C-19 (δ_C_ 43.0 ppm), and from H-3 to C-1 (δ_C_ 48.6 ppm), C-4 (δ_C_ 34.7 ppm), C-4a, C-10 (δ_C_ 169.7 ppm), C-19, C-20 (δ_C_ 30.0 ppm) and C-20′ (δ_C_ 24.1 ppm) supporting the establishment of a 2,4,4-trimethyl-2,3,4,9-tetrahydro-1*H*-β-carboline moiety (**ttbc**, Figure 2). ROESY correlations from NH (δ_H_ 10.62 ppm) to both H-1 and H-8 further confirmed this assignment. A doublet proton at δ_H_ 4.75 ppm (H-12) showed a COSY correlation with an exchangeable proton H-11 (δ_H_ 7.90 ppm) and HMBC correlations to C-12a (δ_C_ 34.3 ppm), C-13 (δ_C_ 171.0 ppm), and C-22 (δ_C_ 26.4 ppm). A tert-butyl group was assigned based on a HMBC correlation from a signal of three equivalent methyls (H-22, δ_H_ 0.90 ppm) to C-12a. These *tert*-butyl protons also showed a HMBC correlation with a methine carbon C-12 at δ_C_ 53.4 ppm indicating the presence of a *tert*-leucine residue (***tert*-Leu**, Figure 2). A spin system =CH-CH-CH-(CH_3_)_2_ was deduced due to COSY correlations from H-16 (δ_H_ 6.59 ppm) to H-15 (δ_H_ 4.89 ppm); from H-15a (δ_H_ 1.91 ppm) to both H-15 and two methyl groups H-24 (δ_H_ 0.77 ppm) and H-24′ (δ_H_ 0.67 ppm). HMBC correlations from H-15 to an *N*-methyl C-23 (δ_C_ 30.8 ppm), H-16 to C-18 (δ_C_ 168.6 ppm) and C-25 (δ_C_ 13.5 ppm) and also from H-25 (δ_H_ 0.67 ppm) to C-16, C-17 (δ_C_ 131.7 ppm) and C-18 suggested the presence of an *N*-methylvinylogous valine residue (**mvv**, Figure 2). The *E*-configuration was assigned to ∆^16−17^ according to the distinctively upfield chemical shift of C-25 at δ_H_ 13–14 ppm (rather than at 22–25 ppm for the *Z*-configuration) [21,22,23]. HMBC correlations from H-12 to C-10, from H-23 to C-13 and ROESY correlations between H-3 and H-11 and also between H-12 and H-23 supported the connection of moieties **ttbc**-***tert*-Leu**-**mvv** (Figure 2). A planar structure of a new milnamide derivative, named milnamide E, was established.

**Table 1:** NMR data for milnamides E, F, and G (**1**–**3**) recorded in DMSO-*d_6_* ^a^ at 30 °C.

Position		1 ^a^								2 ^b^								3 ^b^				
		δ_C_^e^		δ_H_ (*J* in Hz) ^e^		ROESY ^e^	gHMBCAD ^e^			δ_C_^e,f^		δ_H_ (*J* in Hz) ^e^		ROESY ^e^		gHMBCAD ^e^		δ_C_^e^		δ_H_ (*J* in Hz) ^e^	ROESY ^e^	gHMBCAD ^e^
1		48.6		3.62, d (14.4) 3.88, d (14.4)		9 19	3, 4a, 9a 3, 4a, 9a, 19			160.9								156.8		9.14, s	9, 19	3, 4a, 9a, 19
3		72.1		3.40, s		11, 19, 20, 20′	1, 4, 4a, 10, 19, 20			72.5		4.26, s		11, 19, 20, 20′		1, 4, 4a, 10, 19, 20		72.2		4.80, s	11, 19, 20, 20′	1, 4, 4a, 10, 19, 20
4		34.7								35.1								36.6				
4a		113.0								121.9								128.8				
4b		125.4								123.8								122.6				
5		118.6		7.47, d (8.4)		20′	4a, 4b, 7, 8a			120.3		7.61, d (7.8)		20′		7, 8a		122.2		7.80, d (8.4)	20′	7, 8a
6		117.9		6.87, t (7.8)			4b, 8			118.9		6.97, t (7.8)				4b		121.8		7.15, t (7.8)		4b, 8
7		119.5		6.95, t (7.8)			5, 8a			122.8		7.14, t (7.8)				5, 8a		128.3		7.43, t (7.8)		5, 8a
8		110.9		7.24, d (8.4)		9	4b, 6			112.3		7.36, d (7.8)		9		4b		113.8		7.54, d (8.4)	9	4b, 6
8a		135.9								137.0								141.4				
9				10.62, s		1, 8	4a, 4b, 8a, 9a					11.46, s		8		4a, 4b, 8a, 9a				12.36, s	1, 8	4a, 4b, 8a, 9a
9a		131.8								125.6								124.1				
10		169.7								168.7								163.9				
11				7.90, d (9.6)		3, 20′, 22	10					8.22, d (9.0)		3, 20′, 22		10				8.64, d (9.0)	3, 20′, 22	10
12		53.4		4.75, d (9.0)		22, 23	10, 12a, 13, 22			53.7		4.69, d (9.6)		22, 23		12a, 22		55.0		4.64, d (9.0)	22, 23	10, 12a, 13, 22
12a		34.3								34.8								34.9				
13		171.0								170.3								169.8				
15		55.8		4.89, t (10.2)		24, 24′, 25	13, 15a, 16, 17			55.8		4.85, t (10.2)		24, 24′, 25				56.2		4.85, t (10.2)	24, 24′, 25	
15a		28.8		1.91, m		16, 23				28.4		1.88, m		16, 23				28.6		1.87, m	16, 23	
16		138.2		6.59, d (9.6)		15a, 23, 24	15a, 18, 25			138.0		6.59, dd (1.2, 10.2)		15a, 23, 24				138.1		6.59, dd (1.2, 9.6)	15a, 23, 24	18, 25
17	131.7								131.5								131.7					
18	168.6								168.4								168.5					
19	43.0		2.43, s		1, 3, 22			1, 3, 9a	32.6		2.86, s		3, 22		1, 3		45.6		3.58, s		1, 3, 22	1, 3
20	30.0		1.37, s		3			3, 4, 4a, 20′	29.8		1.39, s		3		3, 4, 4a, 20′		29.8		1.40, s		3	3, 4, 4a, 20′
20′	24.1		1.30, s		3, 5, 11			3, 4, 4a, 20	23.1		1.47, s		3, 5, 11		3, 4, 4a, 20		22.1		1.61, s		3, 5, 11	3, 4, 4a, 20
22	26.4		0.90, s		11, 12, 19, 23, 25			12, 12a	26.0		0.91, s		11, 12, 19, 23, 25		12, 12a		26.2		0.94, s		11, 12, 19, 23	12, 12a
23	30.8		2.87, s		12, 15a, 16, 22, 24′			13, 15	30.7		2.81, s		12, 15a, 16, 22, 24′		13, 15		31.0		2.82, s		12, 15a, 16, 22, 24′	13, 15
24	19.3		0.77, d (6.6)		15, 16, 24′			15, 15a, 24′	18.9		0.75, d (6.6)		15, 16, 24′		15, 15a, 24′		19.2		0.73, d (6.6)		15, 16, 24′	15, 15a, 24′
24′	18.8		0.68, d (6.6)		15, 23, 24			15, 15a, 24	18.3		0.58, d (6.6)		15, 23, 24		15, 15a, 24		18.5		0.51, d (6.6)		15, 23, 24	15, 15a, 24
25	13.5		1.76, s		15, 22			16, 17, 18	13.1		1.76, d (1.2)		15, 22		16, 17, 18		13.3		1.77, d (1.2)		16	16, 17, 18
18-OH			^d^								12.40, brs								12.43, brs			

^a^ Compound in a FA form; ^b^ Compound in a TFA form; ^d^ Not determined; ^e^^1^H NMR at 600 MHz referenced to residual DMSO solvent (δ_H_ 2.50 ppm) and ^13^C NMR at 150 MHz referenced to residual DMSO solvent (δ_C_ 39.52 ppm); ^f^^13^C chemical shifts obtained from correlations observed in gHSQCAD and gHMBCAD spectra.

**Figure 3:**
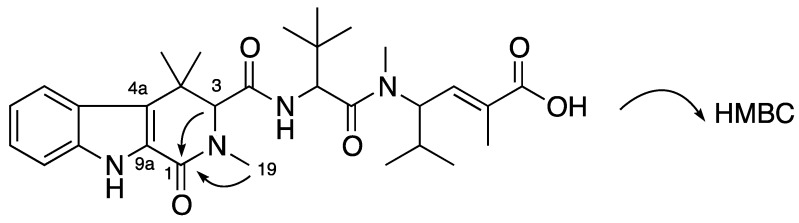
Planar structure of **2** and its key HMBC correlations.

Compound **2** was obtained as a white amorphous solid. The (+)-HRESIMS spectrum showed a signal [M + Na]^+^ ([C_30_H_42_N_4_O_5_Na]^+^) at *m/z* 561.3045 suggesting the molecular formula C_30_H_42_N_4_O_5_. Compared with **1**, **2** lost two protons but gained one oxygen atom together with one additional degree of unsaturation. NMR data of **2** (Table 1) were very similar to those of **1** except the absence of two methylene protons H-1 in the tetrahydro-β-carboline residue. Detailed NMR showed that the methylene at C-1 in **1** was replaced by a carbonyl at δ_C_ 160.9 ppm, which was supported by HMBC correlations from H-3 (δ_H_ 4.26 ppm) and H-19 (δ_H_ 2.86 ppm) to C-1 (Figure 3). The downfield chemical shift of C-4a (δ_C_ 121.9 ppm) confirmed its β position in an α,β-unsaturated carbonyl of a 2,4,4-trimethyl-2,3,4,9-tetrahydro-1*H*-β-carbolinone (Figure 3). On the basis of these data, a planar structure of milnamide F was assigned to **2** (Figure 3).

**Figure 4:**
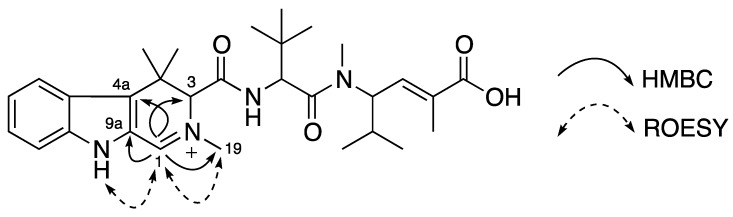
Planar structure of **3** and its key HMBC and ROESY correlations.

Compound **3** was obtained as a yellow amorphous solid. The (+)-HRESIMS spectrum displayed a molecular ion at *m/z* 523.3277 corresponding to the molecular formula of C_30_H_43_N_4_O_4_^+^ with one less proton than that of **1**. NMR data between **3** and **1** were comparable and the loss of one proton was found in the tetrahydro-β-carboline moiety. The two methylene proton signals in **1** were replaced by a one-proton singlet at δ_H_ 9.14 ppm (H-1) corresponding to a downfield sp^2^ methine carbon at δ_C_ 156.8 ppm (C-1). The replacing proton H-1 (δ_H_ 9.14 ppm) showed HMBC correlations to C-3 (δ_C_ 72.2 ppm), C4a (δ_C_ 128.8 ppm), C-9a (δ_C_ 124.1 ppm) and C-19 (δ_C_ 45.6 ppm) and also ROESY correlations to H-9 (δ_H_ 12.36 ppm) and H-19 (δ_H_ 3.58 ppm). The location of the imine carbon C-1 (δ_C_ 156.8 ppm) caused a positive charge at N-2 resulting in the downfield shifts of its neighbor carbons and protons at positions 1, 3, and 19 compared with those in **1** and **2** (Figure 4). Thus, a planar structure of **3** with a 2,4,4-trimethyl-4,9-dihydro-3*H*-β-carboline, named milnamide G, was established as in Figure 4.

We noticed that the dried milnamides A (**5**) and E (**1**) changed from white powders to yellow powders when the compounds were stored at room temperature for a few months. The LC/MS analysis showed about 5%–10% of milnamides D (**7**) or G (**3**) had been formed in milnamides A (**5**) or E (**1**), respectively. Milnamide A (**5**) has previously been reported to be auto-oxidized slowly to milamide D (**7**) while standing in chloroform [24]. As in the previous study, we are unable to determine if milnamides A (**5**) and E (**1**) are naturally occurring or produced by auto-oxidation.

Chemical degradation and subsequent Marfey’s amino acid analysis [25,26,27] revealed that all new compounds **1**–**3** shared the same L configuration of the *tert*-leucine residue with other known milnamides and hemiasterlines (Supplementary Figure S23). Structurally, milnamides E–G (**1**–**3**) differ from milnamides A, C, and D (**5**–**7**) only by the lack of the methyl substituent at N-9. Absolute configurations of milnamides A, C, and D were previously determined by synthesis or X-ray diffraction analysis [8,24]. Therefore, it has been well established that absolute configurations of the new milnamides E–G (**1**–**3**) can be assigned by comparing the signs of Cotton effects (CEs) and specific optical rotations [5,8]. Here the new compound **1** showed similar CEs with milnamide A (**5**) and the new compound **3** showed similar CEs with milnamide D (**7**) (Supplementary Figure S24). Their specific rotation values were also in the same sign (**1**, [α]^25^_D_ +11 (*c* 0.02, MeOH) and **3**, [α]^25^_D_ +134 (*c* 0.04, MeOH)). These data allowed the absolute configurations (3*S*, 12*S*, 15*S*) to be assigned to compounds **1** and **3**. So far, the CD data of milnamide C (**6**) has not been reported. CD spectra of **6** and **2** (Supplementary Figure S24) showed similar CEs indicating that they had similar absolute configuration. The isolated compounds **6** and **2** showed the same positive sign of optical rotation (Supplementary Figure S24) and the same *S-*configuration at C-12 in the *tert*-leucine residue with others milnamide derivatives. Based on biogenetic considerations, the absolute configurations of **6** and **2** were proposed as (3*S*, 12*S*, 15*S*).

This is the first report for the milnamides in which there is an absence of a methyl group on the indolic part. These new milnamides E-G (**1**–**3**) were only identified from the new genus *Pipestela candelabra* while other milnamides A (**5**), C (**6**), and D (**7**) with a methyl group on the indolic part have been reported from other genera *Auletta* sp. [7,8], *Cymbastela* sp. [9], and also from the *P*. *candelabra* in this study.

**Figure 5:**
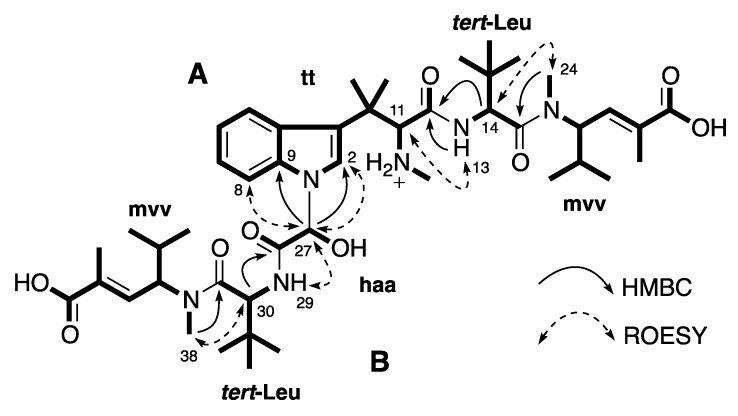
Partial structures **A** and **B** (in bold), and key HMBC and ROESY correlations of **4**.

Compound **4** was obtained as a white amorphous solid. The (+)-HRESIMS showed a signal [M + H]^+^ ([C_46_H_73_N_6_O_9_]^+^) at *m/z* 853.5470 indicating the molecular formula C_46_H_72_N_6_O_9_. NMR analysis revealed that this compound contained six residues including one trimethylated tryptophan (**tt**), two *tert*-leucines (***tert*-Leu**), two *N*-methylvinylogous valines (**mvv**) and one 2-hydroxyacetic acid (**haa**). HMBC correlations and ROESY correlations allowed establishing a tripeptide substructure **A** (**tt-*tert*-Leu-mvv**) (Figure 5), which was identical to the tripeptide structure of hemiasterlin (**8**) and hemiasterlin A (**9**). The remaining atoms in **4** were in a side chain **B** whose sequence (**haa-*tert*-Leu-mvv**) was confirmed by HMBC correlations from H-30 (δ_H_ 4.73 ppm) to C-28 (δ_C_ 167.6 ppm), H-38 (δ_H_ 2.94 ppm) to C-31 (δ_C_ 170.3 ppm) (Figure 5). The observation of HMBC correlations from H-27 (δ_H_ 6.34 ppm) to C-2 (δ_C_ 125.2 ppm) and C-9 (δ_C_ 136.5 ppm) supported the linkage between **A** and **B** at an indole nitrogen N-1 and C-27 (Figure 5). This connection was supported by ROESY correlations from H-27 to H-2 (δ_H_ 7.20 ppm) and H-8 (δ_H_ 7.54 ppm). A planar structure of a new hemiasterlin, hemiasterlin D (**4**), was determined as in Figure 5.

The presence of two l-*tert*-Leu residues in **4** was established since only a single peak corresponding to l-*tert*-Leu was observed in the ion mass extraction chromatogram of its Marfey’s derivatives (Supplementary Figure S23). Compound **4** showed the same sign of optical rotation with our isolated hemiasterlin (**8**) ([α]^25^_D_ −73 (*c* 0.06, MeOH), lit. [α]^25^_D_ −95 (*c* 0.06, MeOH) [4]), hemiasterlin A (**9**) ([α]^25^_D_ −60 (*c* 0.25, MeOH), lit. [α]^25^_D_ −45 (*c* 0.25, MeOH) [5]) and hemiasterlin C ([α]^25^_D_ −18.8 (*c* 0.11, MeOH) [6]) (literature did not report the optical value of hemiasterlin B [5]). On the basis of biogenetic considerations, the two *N*-methylvinylogous valines and the trimethylated tryptophan residue of **4** were proposed to have an *S*-configuration as those in the other hemiasterlin analogues. Hemiasterlin D is the first hemiasterlin with a peptide side chain containing 2-hydroxyacetic acid, *tert*-leucine and *N*-methylvinylogous valine residues at N-1.

The remaining eight known compounds including milnamide A (**5**) [7,9], milnamide C (**6**) [8], milnamide D (**7**) [8,9], hemiasterlin (or milnamide B) (**8**) [4,5,6,8,9], hemiasterlin A (**9**) [5,6], geodiamolide D (**10**) [19,20], geodiamolide E (**11**) [19,20], and geodiamolide F (**12**) [19,20] were all identified by comparisons of the NMR, MS and optical rotation values with those in the literature.

Antiproliferative effect of isolated compounds was evaluated against the human prostate cancer cell line (PC3) and the human neonatal foreskin fibroblast non-cancer cell line (NFF). All compounds showed potent inhibition against prostate cancer cells with IC_50_ values from the picomolar to sub-micromolar range (Table 2). Selective indexes between PC3 cells and NFF cells were from 2.6 to 8.3 indicating these compounds had some selective activity towards prostate cancer cells. Cytotoxic results also indicated that breaking the β-carboline system in milnamides to form the tryptophan system in hemiasterlins increased cytotoxicity.

In the milnamide family, milnamide A (**5**) exhibited the strongest activity (IC_50_ = 11.0 nM) and was 3-fold more potent than milnamide C (**6**) and 35-fold more potent than milnamide C (**7**). A similar trend was observed in the activity of milnamide E to G (**1**–**3**). The results indicated that replacing the methylene at C-1 with a carbonyl or an imine led to a decrease in cytotoxicity. Losing the *N*-methyl at *N*-9 in the β-carboline ring converting **5** to **1**, **6** to **2**, and **7** to **3** also reduced the activity. These data suggested that the *N*-methyl at N-9 might contribute to the milnamide cytotoxicity.

**Table 2:** Cytotoxic evaluation for compounds **1**–**12**.

Compound	IC50 (nM) or% Inhibition ^a^	
	PC3	NFF
**1**	34.2	123
**2**	2180	5650
**3**	2870	39% at 10 μM
**4**	2.20	8.16
**5**	11.0	70.6
**6**	31.7	188
**7**	381	1190
**8**	0.0484	0.404
**9**	0.269	1.03
**10**	33.1	99.1
**11**	118	425
**12**	155	708
**Taxol**	2.26	3.94
**Doxorubicin**	359	530

^a^ Each IC_50_ (nM) or % inhibition at 10 μM was determined as the mean of two independent experiments with triplicate determinations for each concentration.

Compared with milnamides (**1**–**3** and **5**–**7**), hemiasterlins (**4**, **8**, and **9**) demonstrated much more potent cytotoxicity and showed higher selectivity towards noncancer cells. Their activity against the PC3 cells was in the picomolar to sub-nanomolar range. In the examination of hemiasterlins, Andersen *et al*. [10] found that the *N*-methyl indole had better activity than the NH indole. Here a methyl at the indole nitrogen N-1 in hemiasterlin (**8**) was found six-fold more cytotoxic than an NH indole in hemiastermin A (**9**). This cytotoxicity result was comparable with those reported for other cancer cell lines [5,6]. In our study, the new compound, hemiasterlin D (**4**) showed inhibition with IC_50_ values of 2.20 and 8.16 nM for PC3 and NFF cells, respectively. Although the new hemiasterlin D (**4**) here was less potent than the known analogues, the results showed that cytotoxicity was maintained when replacing the methyl of the *N*-methylindole in the tetramethyltryptophan residue with a long side chain. This result may offer a new position at N-1 for hemiasterlin to be developed for more selective activity and better pharmaceutical properties.

The three depsipeptides, geodiamolides D–F (**10**–**12**), which were found co-occurring with the milnamides and hemiasterlins, also displayed strong cytotoxic activity against the PC3 cells and showed 3- to 5-fold selectivity with the NFF cells. Geodiamolide D (**10**) showed the strongest activity with an IC_50_ value of 33.1 nM, which was 4-fold more potent than geodiamolide E (**11**) and 5-fold more active than geodiamolide F (**12**). This data indicated that there was a difference in cytotoxicity due to the halogen atoms attached to the geodiamolide skeleton. The cytotoxic trend of geodiamolides D–F (**10**–**12**) against PC3 cells decreased from iodine to bromine and chlorine. This trend in the human PC3 cell line was totally different from the previous cytotoxic trend reported for these compounds in the murine leukemia L1210 cell line (IC_50_ values of 62.2, 24.2, and 11.2 nM for geodiamolides D, E and F, respectively) [19]. How these halogen atoms influence cytotoxicity is something that warrants further investigation.

## 3. Experimental Section

### 3.1. General Experimental Procedures

Optical rotations were measured on JASCO P-1020 polarimeter (10 cm cell). Circular dichroism spectra were measured on JASCO J-715 Spectropolarimeter Circular Dichroism/Optical Rotatory Dispersion. UV spectra were recorded on a CAMSPEC M501 UV/VIS spectrophotometer. NMR spectra were recorded at 30 °C on a Varian Inova 600 MHz spectrometer. The ^1^H and ^13^C chemical shift were referenced to the DMSO-*d_6_* solvent peak at δ_H_ 2.50 and δ_C_ 39.52 ppm. Standard parameters were used for the 2D NMR spectra obtained, which included gCOSY, gHSQCAD (^1^*J*_CH_ = 140 Hz), gHMBCAD (*^n^J*_CH_ = 8 Hz) and ROESY. Mass spectra were acquired using a Mariner TOF mass spectrometer (Applied Biosystems Pty Ltd.). High-resolution mass measurement was acquired on a Bruker Solarix 12 Tesla Fourier transform mass spectrometer, fitted with an Apollo API source. For the HPLC isolation, a Water 600 pump equipped with a Water 966 PDA detector and Gilson 715 liquid handler were used. A Betasil C_18_ column (5 μm, 21.2 × 150 mm) and Hypersil BDS C_18_ column (5 μm, 10 × 250 mm) were used for semipreparative HPLC. A Phenomenex Luna C_18_ column (3 μm, 4.6 × 50 mm) was used for LC/MS controlled by MassLynx 4.1 software. All solvents used for extraction and chromatography were HPLC grade from RCI Labscan or Burdick & Jackson, and the H_2_O used was ultrapure water (Arium^®^ proVF) from Sartorius Stedim Biotech.

### 3.2. Animal Material

A first sample of *P*. *candelabra* was collected at the depth of 36 m, Wilson Reef, Coral Sea, Queensland, Australia. It was identified as *Pipestela candelabra* (phylum Porifera, class Demospongiae, order Halichondrida, family Axinellidae). A voucher specimen QMG320579 has been deposited at the Queensland Museum, South Brisbane, Queensland, Australia.

A second sample characterised as the same taxonomy with the first specimen *Pipestela candelabra* was collected at the depth of 20 m, Houghton Reef, Howick Group, Queensland, Australia. A voucher specimen QMG320790 has been deposited at the Queensland Museum, South Brisbane, Queensland, Australia.

### 3.3. Extraction and Isolation

A first freeze-dried sample of *P*. *candelabra* (19 g) collected at Wilson Reef, Coral Sea was extracted exhaustively with hexane (250 mL), dichloromethane (DCM) (3 × 250 mL) and MeOH (3 × 250 mL), respectively. The DCM and MeOH extracts were combined and then evaporated solvents to yield a yellow residue (2.6 g). This crude extract was pre-adsorbed onto C_18_ (1.0 g) and packed dry into a small cartridge, which was connected to a C_18_ preparative HPLC column (5 μm, 21.2 × 150 mm). A linear gradient from 100% H_2_O (0.1% TFA) to 100% MeOH (0.1% TFA) was performed over 60 min at a flow rate of 9.0 mL/min and 60 fractions (1.0 min each) were collected. Fractions 39–40 were combined and loaded on the Hypersil BDS C_18_ column (5 μm, 10 × 250 mm) with a linear gradient from 60% MeOH (0.1% TFA)–40% H_2_O (0.1% TFA) to 100% MeOH (0.1% TFA) at a flow rate of 4 mL/min in 60 min yielding **6** (1.2 mg, 0.006% dry wt). Fractions 30–38 were combined and chromatographed on the Betasil C_18_ column (5 μm, 21.2 x 150 mm) from 50% MeOH (0.1% TFA)–50% H_2_O (0.1% TFA) to 100% MeOH (0.1% TFA) in 60 min. Three pure compounds were isolated including **7** (2.5 mg, 0.013% dry wt), **8** (4 mg, 0.021% dry wt), and **9** (5 mg, 0.026% dry wt). Fraction 47 was further purified on the Hypersil BDS C_18_ column (5 μm, 10 × 250 mm) with a linear gradient from 40% MeOH (0.1% TFA)–60% H_2_O (0.1% TFA) to 45% MeOH (0.1% TFA)–55% H_2_O (0.1% TFA) at a flow rate of 4 mL/min in 45 min yielding **3** (1.5 mg, 0.008% dry wt) and **7** (0.5 mg, 0.003% dry wt). Fraction 50 was loaded on the Hypersil BDS C_18_ column (5 μm, 10 × 250 mm) with a linear gradient from 45% MeOH (0.1% TFA)–55% H_2_O (0.1% TFA) to 55% MeOH (0.1% TFA)–45% H_2_O (0.1% TFA) at a flow rate of 4 mL/min in 60 min resulting in the purification of compounds **1** (0.8 mg, 0.004% dry wt) and **5** (1 mg, 0.005% dry wt). Fraction 53 was also subjected on the Hypersil BDS C_18_ column (5 μm, 10 × 250 mm) with a linear gradient from 50% MeOH (0.1% TFA)–50% H_2_O (0.1% TFA) to 100% MeOH (0.1% TFA) at a flow rate of 4 mL/min in 60 min to obtain **10** (3.1 mg, 0.016% dry wt) and **11** (2.1 mg, 0.011% dry wt). Fractions 55–56 were also loaded on the Hypersil BDS C_18_ column (5 μm, 10 × 250 mm) with a linear gradient from 50% MeOH (0.1% TFA)–50% H_2_O (0.1% TFA) to 65% MeOH (0.1% TFA)–35% H_2_O (0.1% TFA) at a flow rate of 4 mL/min in 60 min yielding **2** (0.9 mg, 0.005% dry wt) and **12** (0.5 mg, 0.003% dry wt).

A second freeze-dried sample of *P*. *candelabra* (30 g) collected at Houghton Reef, Howick Group, was also extracted exhaustively with hexane (750 mL), DCM (4 × 250 mL) and MeOH (4 × 250 mL), respectively. The DCM and MeOH extracts were combined and then evaporated solvents to yield a yellow residue (7.5 g). This crude extract was pre-adsorbed onto C_18_ (10 g) and packed dry into a cartridge (25 × 50 mm), which was connected to a C_18_ preparative HPLC column (5 μm, 50 × 150 mm). A linear gradient from 100% H_2_O (0.1% TFA) to 100% MeOH (0.1% TFA) was performed over 90 min at a flow rate of 20 mL/min and 180 fractions (0.5 min each) were collected. Fractions 111 to 114 were then combined and further purified by a Betasil C_18_ column (5 μm, 21.2 × 150 mm) using a linear gradient from 45% MeOH (0.1% TFA)–55% H_2_O (0.1% TFA) to 100% MeOH (0.1% TFA) in 60 min. Compounds **7** (1.0 mg, 0.0033% dry wt), **9** (12.0 mg, 0.0400% dry wt) and **4** (0.8 mg, 0.0026% dry wt) were obtained. A 15 mg mixture of compounds **5** and **1** in fractions 115 to 124 was subjected on the Hypersil BDS C_18_ column (5 μm, 10 × 250 mm) with a 60 min linear gradient from 50% MeOH (0.1% FA)–50% H_2_O (0.1% FA) to 100% MeOH (0.1% FA) yielding **1** (2.5 mg, 0.0083% dry wt) and **5** (2.0 mg, 0.0067% dry wt). The combined fractions 125 to 132 were also chromatographed on the Betasil C_18_ column (5 μm, 21.2 × 150 mm) from 50% MeOH (0.1% TFA)–50% H_2_O (0.1% TFA) to 100% MeOH (0.1% TFA) in 60 min. Six compounds were purified, including compounds **8** (18.0 mg, 0.06% dry wt), **12** (1.0 mg, 0.0033% dry wt), **11** (7.0 mg, 0.0233% dry wt), **10** (15.0 mg, 0.05% dry wt) and **6** (0.5 mg, 0.0017% dry wt), respectively.

**Milnamide E (1):** white amorphous solid; [α]^25^_D_ +11 (*c* 0.02, MeOH); CD (MeOH) λ_max_ (∆ε) 245 (−3.9), 266 (+0.38) nm; UV (MeOH) λ_max_ (logε) 227 (4.3), 290 (3.4) nm; ^1^H (600 MHz) and ^13^C (150 MHz) NMR data are summarized in Table 1; (+) HRESIMS *m/z* 525.3418 ([M + H]^+^) (calcd (+) *m/z* 525.3435, ∆ −3.2 ppm).

**Milnamide F (2):** white amorphous solid; [α]^25^_D_ +29 (*c* 0.05, MeOH); CD (MeOH) λ_max_ (∆ε) 241 (−1.2), 260 (+3.1), 329 (+2.6) nm; UV (MeOH) λ_max_ (logε) 210 (3.6), 306 (3.1) nm; ^1^H (600 MHz) and ^13^C (150 MHz) NMR data are summarized in Table 1; (+) HRESIMS *m/z* 561.3045 ([M + Na]^+^) (calcd (+) *m/z* 561.3047, ∆ −0.36 ppm).

**Milnamide G (3):** yellow amorphous solid; [α]^25^_D_ +134 (*c* 0.04, MeOH); CD (MeOH) λ_max_ (∆ε) 240 (−1.96), 284 (+0.58), 371 (−1.4), 438 (+5.0) nm; UV (MeOH) λ_max_ (logε) 207 (4.1), 378 (3.7) nm; ^1^H (600 MHz) and ^13^C (150 MHz) NMR data are summarized in Table 1; (+) HRESIMS *m/z* 523.3277 ([M + H]^+^) (calcd (+) *m/z* 523.3279, ∆ −0.38 ppm).

**Hemiasterlin D (4):** white amorphous solid; [α]^25^_D_ −52 (*c* 0.02, MeOH); UV (MeOH) λ_max_ (logε) 225 (4.3), 270 (3.3) nm; ^1^H (600 MHz) and ^13^C (150 MHz) NMR data are summarized in Table 3; (+) HRESIMS *m/z* 853.5470 ([M + H]^+^) (calcd (+) *m/z* 853.5434, ∆ 4.2 ppm).

**Table 3:** NMR data for TFA salt of hemiasterlin D (**4**) recorded in DMSO-*d_6_* ^a^ at 30 °C.

Position	δ_C_^b^	δ_H_ (*J* in Hz)	gCOSY	ROESY	gHMBCAD
2	125.2	7.20, s		22, 27	3, 4, 9, 10, 27
3	118.0				
4	125.4				
5	120.2	8.11, d (7.2)	6	11, 22	7, 9
6	119.0	7.11, t (7.2, 7.8)	5, 7		4, 8
7	121.2	7.15, t (7.2, 7.8)	6, 8		5, 9
8	110.8	7.54, d (8.4)	7	27	4, 6
9	136.5				
10	37.6				
11	66.9	4.47, d (10.2)	NH	5, 13, 21, 22	
12	165.9				
13		8.90, d (7.8)	14	11, 23	12
14	55.4	4.87, d (7.8)	13	24, 23	12, 14a, 15, 23
14a	34.5				
15	169.9				
17	56.0	4.93, t (9.6, 10.2)	17a, 18	25, 25′, 26	17a, 18, 19
17a	28.6	2.03, m	17, 25, 25′	18, 24	
18	138.0	6.68, d (9.6)	17	24, 17a, 25	20, 26
19	131.7				
20	168.4				
21	33.3	2.24, t (4.2)	NH	11, 23	11
22	26.9	1.37, s		5, 11	3, 10, 11, 22′
22′	21.9	1.38, s		2	3, 10, 11, 22
23	26.1	1.01 (s)		14, 24	14, 14a
24	31.0	3.04 (s)		14, 17a, 18, 23	15, 17
25	19.0	0.82, d (6.0)			17, 17a, 25′
25′	19.0	0.81, d (6.0)			17, 17a, 25
26	13.3	1.80, s		17	18, 19, 20
27	76.3	6.34, s		2, 8, 29	2, 9, 28
28	167.6				
29		8.10, d (8.4)	30	27, 37	28
30	53.9	4.73, d (9.0)	29	37, 38	28, 30a, 31, 37
30a	34.8				
31	170.3				
33	55.5	5.01, t (9.6, 10.2)	33a, 34	39, 39′, 40	31, 33a, 34, 35, 38
33a	28.5	1.97, m	33, 39, 39′	34, 38	
34	137.8	6.64, d (9.6)	33	33a, 38	36, 40
35	132.2				
36	168.3				
37	25.8	0.88, s		29, 30, 38, 40	30, 30a
38	30.4	2.94, s		30, 33a, 34, 37	31, 33
39	18.7	0.79, d (6.6)			33, 33a, 39′
39′	18.7	0.74, d (6.6)			33, 33^a^, 39
40	13.3	1.80, s		33	34, 35, 36
11-NH		8.88, brs 7.51, brs	11, 21 21		
20-OH/36-OH		12.4, brs			

^a^ ^1^H NMR at 600 MHz referenced to residual DMSO solvent (δ_H_ 2.50 ppm) and ^13^C NMR at 150 MHz referenced to residual DMSO solvent (δ_C_ 39.52 ppm); ^b^ ^13^C chemical shifts obtained from correlations observed in gHSQCAD and gHMBCAD spectra.

### 3.4. Peptide Hydrolysis and LC/MS Analysis of Marfey’s Derivatives

Peptide samples (150 μg) were dissolved in degassed 6 N HCl (500 μL) and heated at 120 °C for 8 h. The solvent was removed under dry nitrogen and the resulting material was subjected to further derivatization for a stereochemical assignment.

The hydrolysate mixture or the amino acid standard was added a solution of l-FDAA 1% (w/w) in acetone and 100 μL of a 1 N NaHCO_3_ solution. The vial was heated at 50 °C for 3 h and the contents were neutralized with 2 N HCl (50 μL) after cooling to room temperature. An aliquot of the l-FDAA derivative was dried under dry nitrogen, diluted in MeOH and loaded on a Phenomenex Luna column (C_18_, 3 μm, 4.6 mm × 50 mm) using a linear gradient from 5% MeOH (0.1% FA)–95% H_2_O (0.1% FA) to 100% MeOH (0.1% FA) in 12 min. FDAA derivatives were detected by absorption at 340 nm and assignment was secured by ion mass extraction.

Retention times of authentic FDAA-amino acids are given in parenthesis: l-*tert*-Leu (8.66 min) and d-*tert*-Leu (9.33 min). The hydrolysate of milnamide E (**1**), milnamide F (**2**), milnamide G (**3**), and hemiasterlin D (**4**) contained l-*tert*-Leu eluted at 8.65, 8.72, 8.67, and 8.68 min, respectively (Supplementary Figure S23).

### 3.5. Cytotoxicity Assay

Human prostate adenocarcinoma cells (PC3) and human neonatal foreskin fibroblast (noncancer cells, NFF) were grown in media RPMI-1640 (Life Technologies, Grand Island, NY, USA) supplemented with 10% foetal bovine serum (FBS). Cells were grown under 5% CO_2_ in a humidified environment at 37°C. Fifty microlitres of media containing 500 cells were added to a 384 well microtitre plate (Perkin Elmer, Waltham, MA, USA, part number: 6007660) containing 0.2 μL of a compound. Final compound concentration range tested was 10 μM to 1 pM (final DMSO concentration of 0.4%). Each concentration in media was tested in triplicate and in two independent experiments. Cells and compounds were then incubated in 72 h at 37 °C, 5% CO_2_ and 80% humidity. Cell proliferation was measured with the addition of 10 μL of a 60% Alamar blue (Invitrogen, Carlsbad, CA, USA) solution in media to each well of the microtitre plate to give a final concentration of 10% Alamar blue. The plates were incubated at 37 °C, 5% CO_2_ and 80% humidity within 24 h. The fluorescence of each well was read at excitation 535 nm and emission 590 nm on the Perkin Elmer EnVision Multilabel Reader 2104. Eight-point concentration response curves were then analysed using non-linear regression and IC_50_ values determined by using GraphPad Prism 5 (GraphPad Software Inc., La Jolla, CA, USA). Paclitaxel and doxorubicin were used during each screening as positive control compounds.

## 4. Conclusions

Contributing to the chemical investigation of the new sponge genus *Pipestela candelabra*, twelve secondary metabolites belonging to three structure classes including milnamide, hemiasterlin and geodiamolide were isolated. Three new members of the milnamide family, milnamides E–G (**1**–**3**), and one new member of the hemiasterlin family, hemiasterlin D (**4**), were identified. Milnamides F (**2**) and G (**3**) were identified in the sponge collected at Wilson Reef while hemiasterlin D was only discovered in the sample collected at Houghton Reef. However, all three structure classes were found in samples of the *P*. *candelabra* collected at two locations which are 124 km apart. 

All new and known natural products showed potent and selective inhibition against the human prostate cancer cells (PC3). Hemiasterlin D (**4**) is the first example of a hemiasterlin substituted at N-1 by a peptide side chain, which is identical to the peptide side chain at N-13. The compound has less cytotoxicity (IC_50_ of 2.20 nM) compared to hemiasterlin (**8**) and hemiasterlin A (**9**) (IC_50_ of 0.0484 nM and 0.269 nM, respectively) but it opens the possibility for further structural variation at N-1. This is a first report of the preliminary structure-activity relationships of the milnamide family. The C-1 and N-9 in milnamides are important positions affecting their cytotoxicity in PC3 cells.

## Supplementary Files

Supplementary File 1 Supplementary Information (PDF, 3596 KB)
