# Supplementary material for: Potent Cytotoxic Peptides from the Australian Marine Sponge Pipestela candelabra
Source: Mar Drugs. 2014 Jun 4;12(6):3399–415. doi: 10.3390/md12063399 (PMC4071583; doi:10.3390/md12063399)
Supplement: Supplementary File 1 — Supplementary Information (PDF, 3596 KB) [file marinedrugs-12-03399-s001.pdf]

Supplementary Information

**Figure S1**	^{1}H NMR Spectrum for milnamide E (**1**) in DMSO-d_6
**Figure S2**	^{13}C NMR Spectrum for milnamide E (**1**) in DMSO-d_6
**Figure S3**	gHSQCAD Spectrum for milnamide E (**1**) in DMSO-d_6
**Figure S4**	gHMBCAD Spectrum for milnamide E (**1**) in DMSO-d_6
**Figure S5**	gCOSY Spectrum for milnamide E (**1**) in DMSO-d_6
**Figure S6**	ROESY Spectrum for milnamide E (**1**) in DMSO-d_6
**Figure S7**	^{1}H NMR Spectrum for milnamide F (**2**) in DMSO-d_6
**Figure S8**	gHSQCAD Spectrum for milnamide F (**2**) in DMSO-d_6
**Figure S9**	gHMBCAD Spectrum for milnamide F (**2**) in DMSO-d_6
**Figure S10**	gCOSY Spectrum for milnamide F (**2**) in DMSO-d_6
**Figure S11**	ROESY Spectrum for milnamide F (**2**) in DMSO-d_6
**Figure S12**	^{1}H NMR Spectrum for milnamide G (**3**) in DMSO-d_6
**Figure S13**	gHSQCAD Spectrum for milnamide G (**3**) in DMSO-d_6
**Figure S14**	gHMBCAD Spectrum for milnamide G (**3**) in DMSO-d_6
**Figure S15**	gCOSY Spectrum for milnamide G (**3**) in DMSO-d_6
**Figure S16**	ROESY Spectrum for milnamide G (**3**) in DMSO-d_6
**Figure S17**	^{1}H NMR Spectrum for hemiasterlin D (**4**) in DMSO-d_6
**Figure S18**	gHSQCAD Spectrum for hemiasterlin D (**4**) in DMSO-d_6
**Figure S19**	gHMBCAD Spectrum for hemiasterlin D (**4**) in DMSO-d_6
**Figure S20**	gCOSY Spectrum for hemiasterlin D (**4**) in DMSO-d_6
**Figure S21**	ROESY Spectrum for hemiasterlin D (**4**) in DMSO-d_6
**Figure S22**	(+)-HRESIMS Spectrum for hemiasterlin D (**4**)
**Figure S23**	LC/MS Analysis of Marfey's Derivatives of compounds **1**–**4**
**Figure S24**	CD Spectra and Specific Rotations of Compounds **1**–**3** and **5**–**7**
**Figure S25**	Photographs of the sponges *Pipestela candelabra*

Figure S1. ^{1}H NMR Spectrum for milnamide E (**1**) in DMSO-d_6.

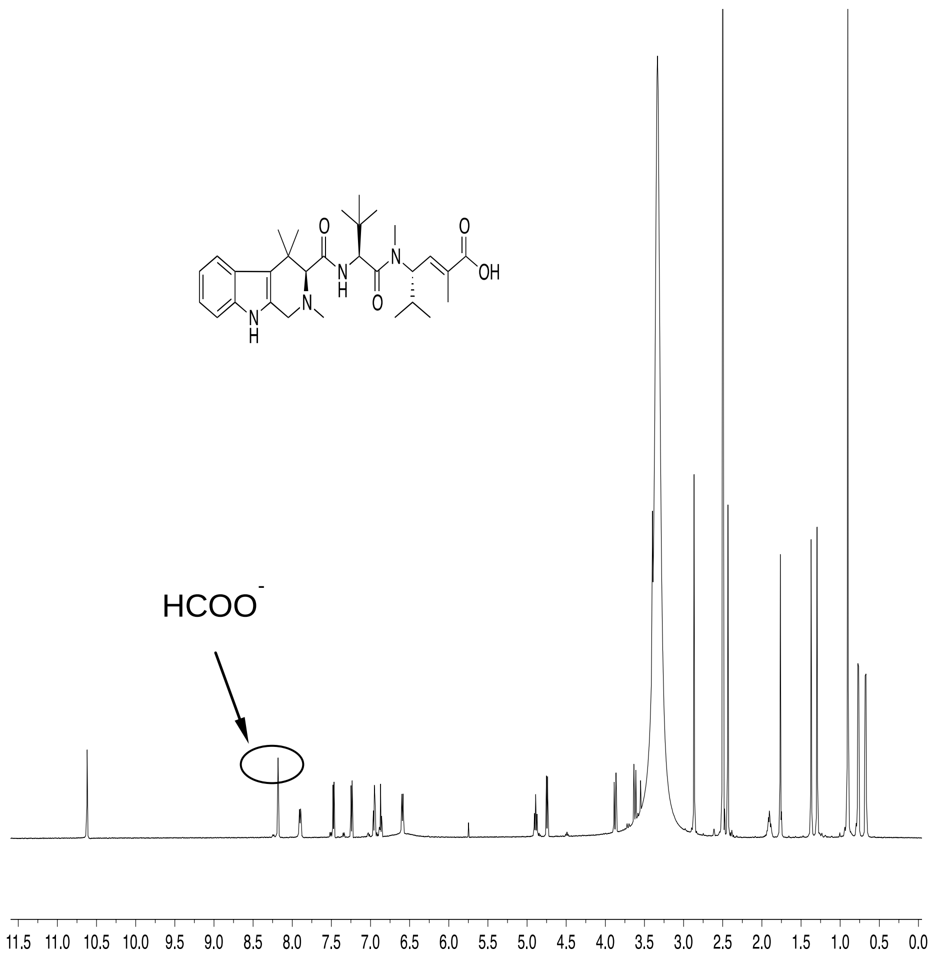

Figure S2. ^{13}C NMR Spectrum for milnamide E (**1**) in DMSO-d_6.

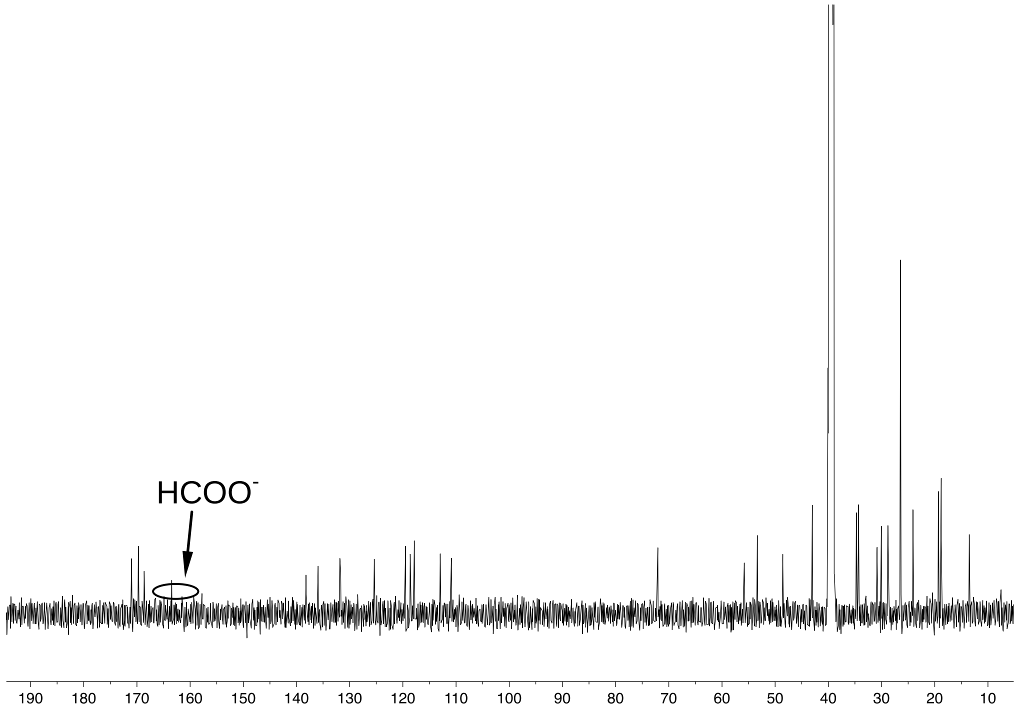

Figure S3. gHSQCAD Spectrum for milnamide E (**1**) in DMSO-d_6.

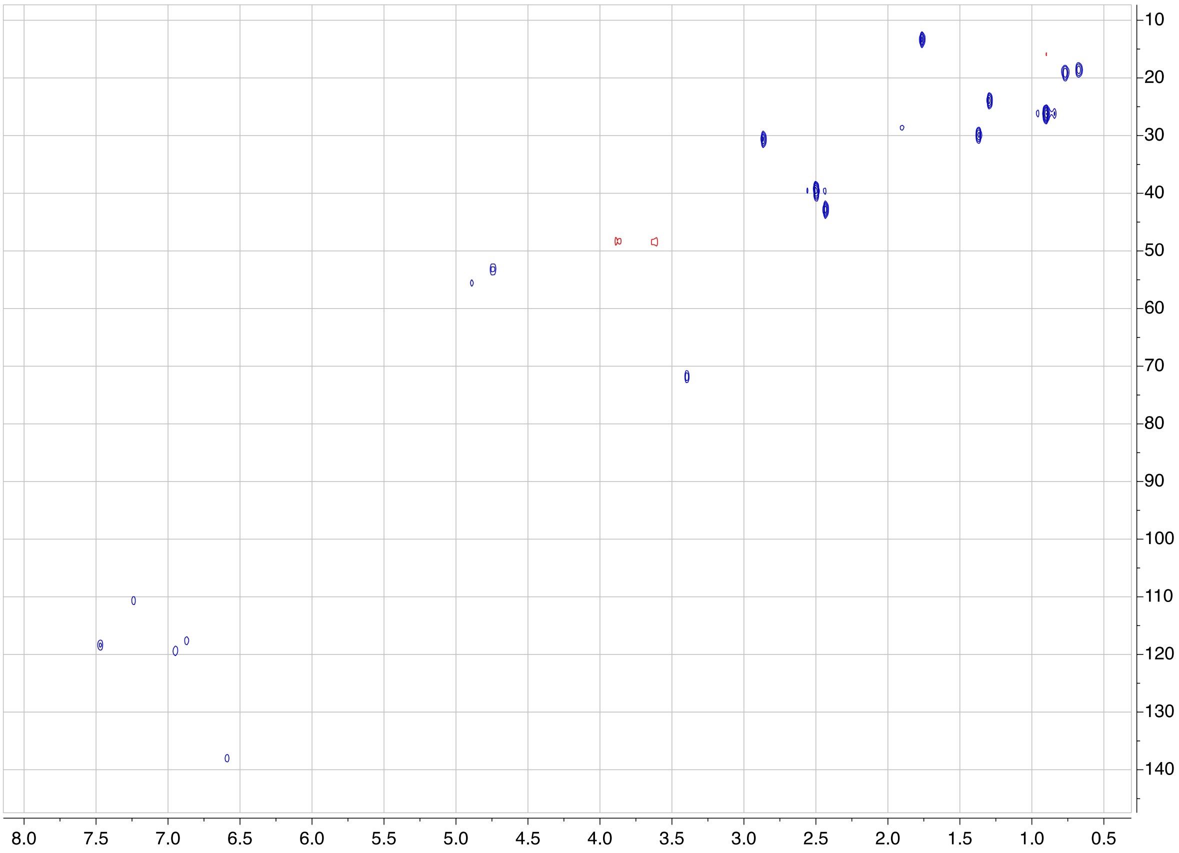

Figure S4. gHMBCAD Spectrum for milnamide E (**1**) in DMSO-d_6.

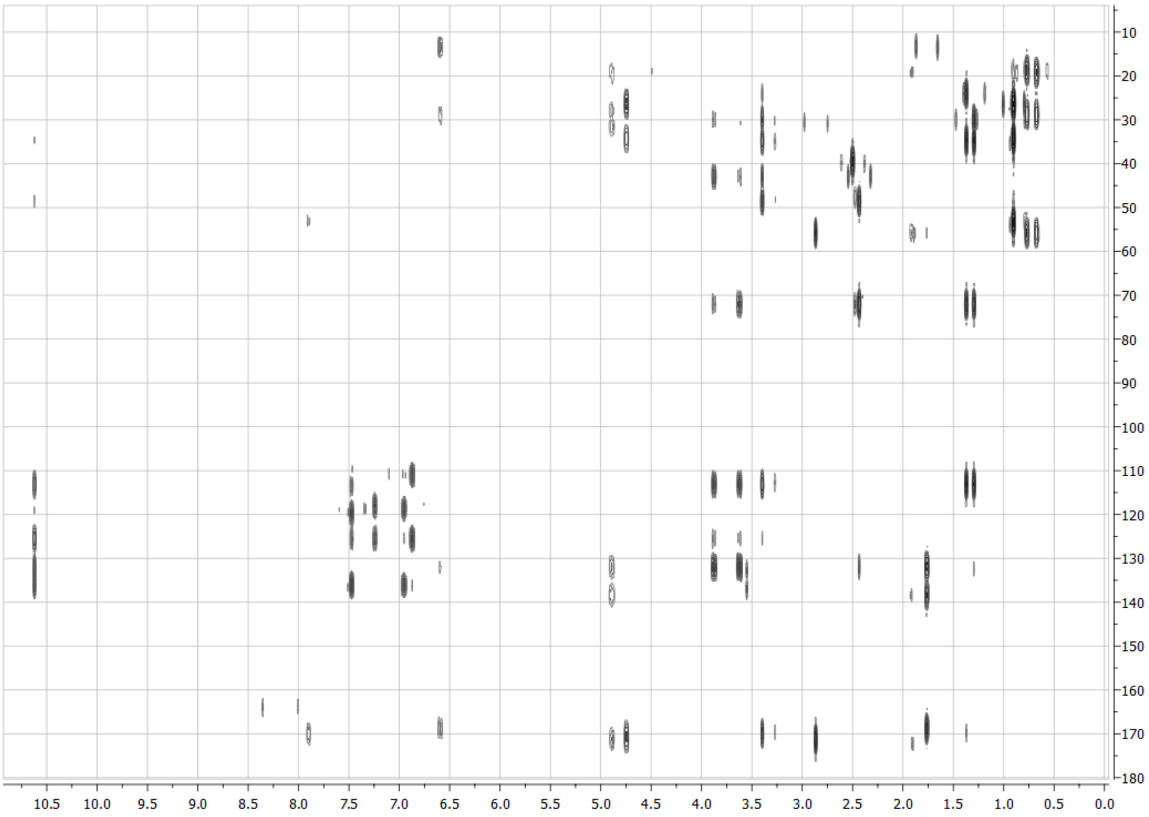

Figure S5. gCOSY Spectrum for milnamide E (**1**) in DMSO-d_6.

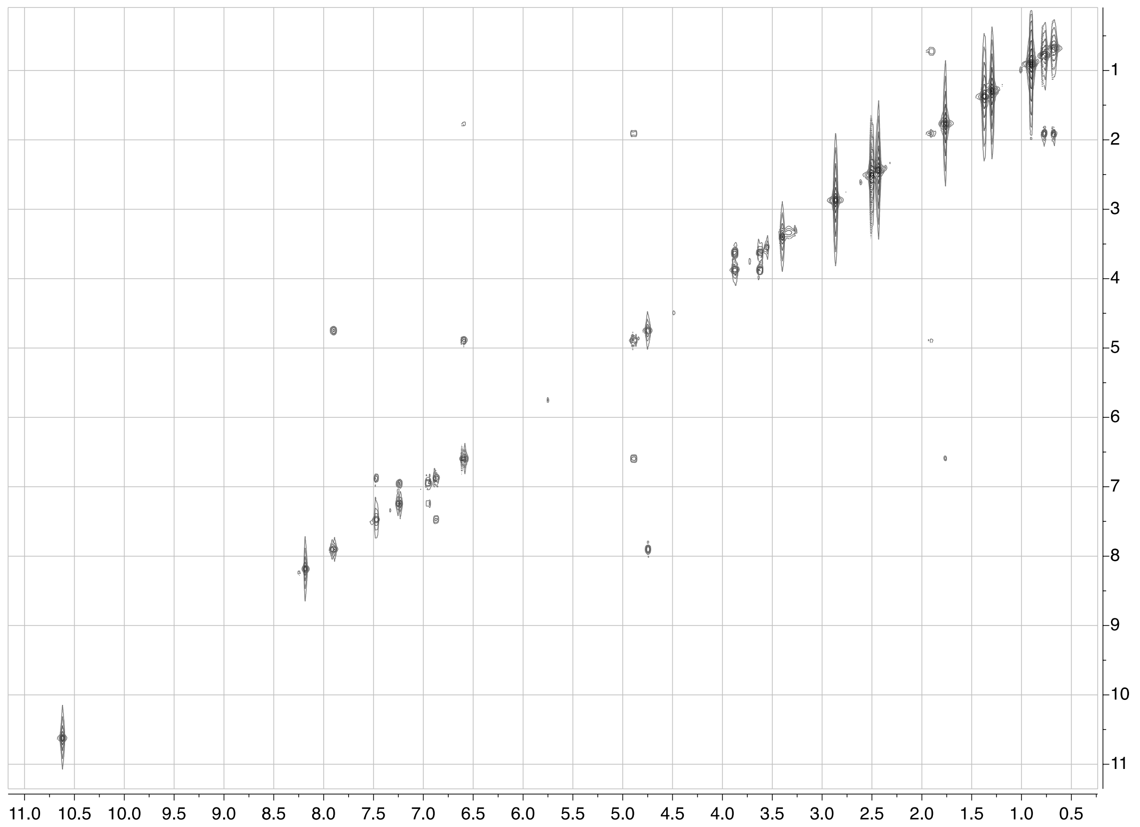

Figure S6. ROESY Spectrum for milnamide E (**1**) in DMSO-d_6.

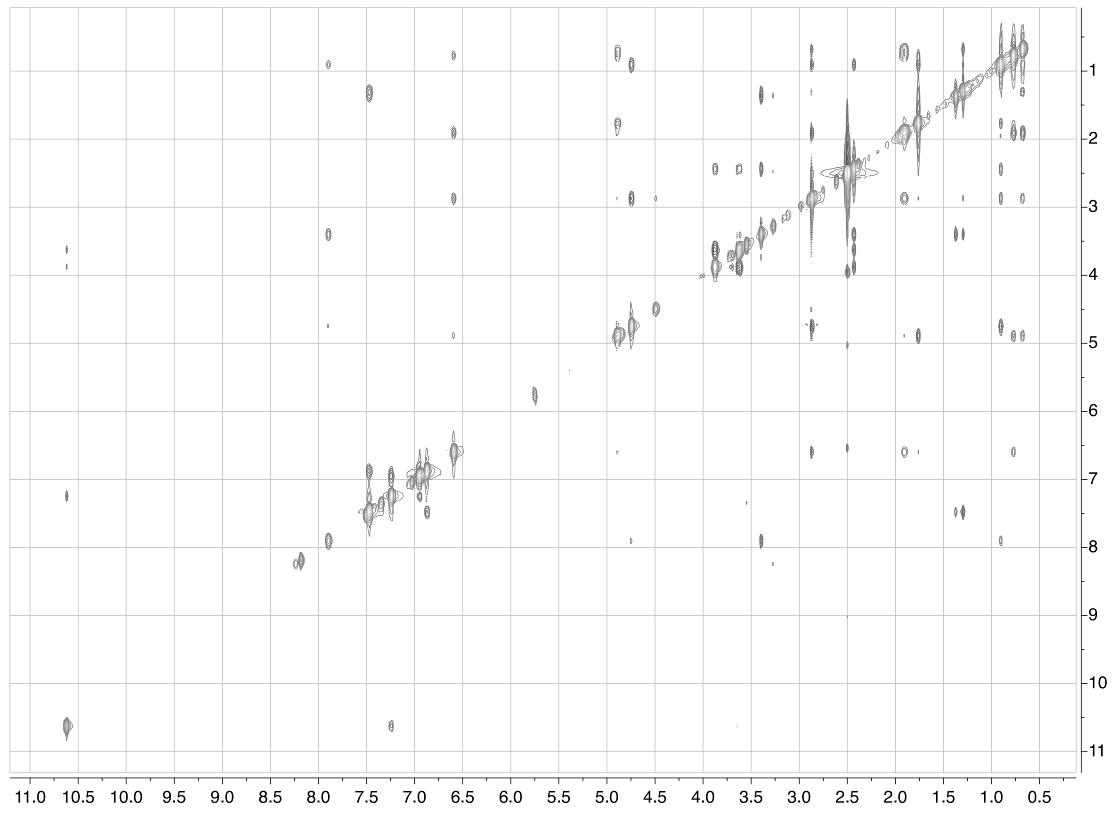

Figure S7. ^{1}H NMR Spectrum for milnamide F (**2**) in DMSO-d_6.

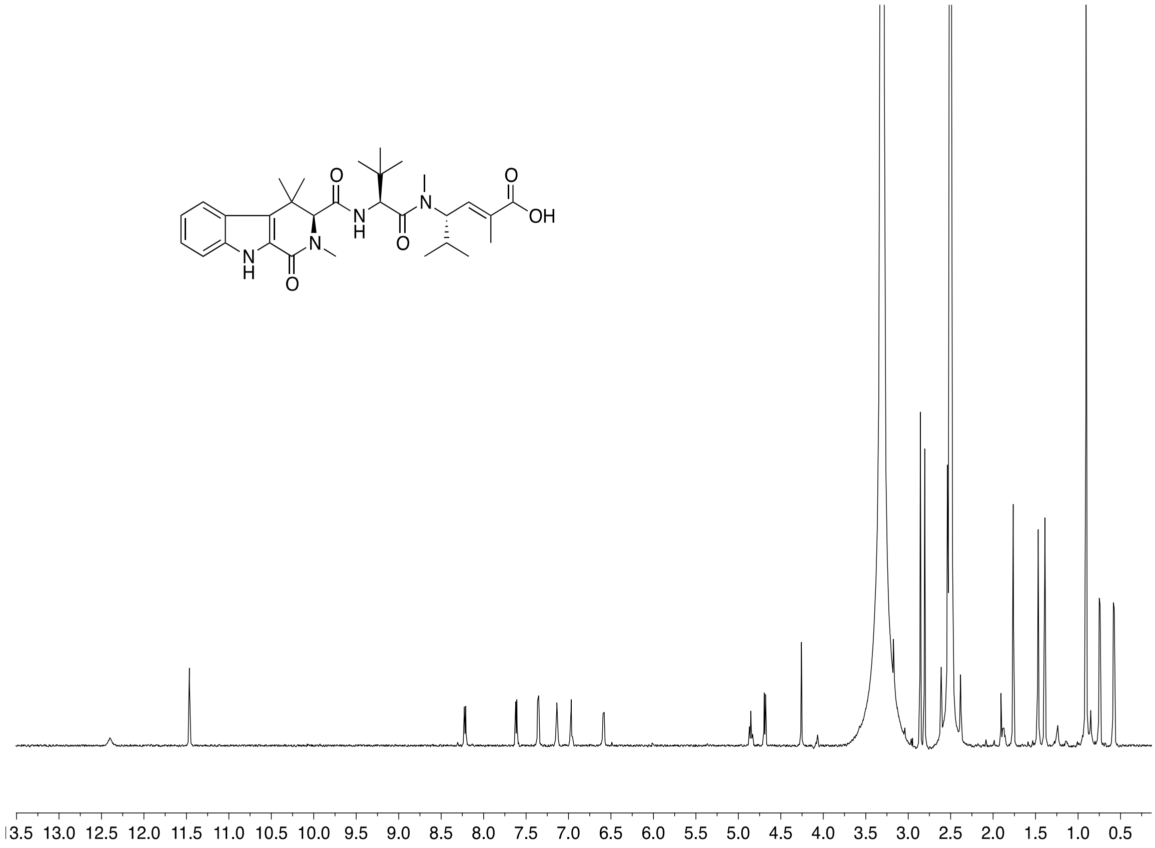

Figure S8. gHSQCAD Spectrum for milnamide F (**2**) in DMSO-d_6.

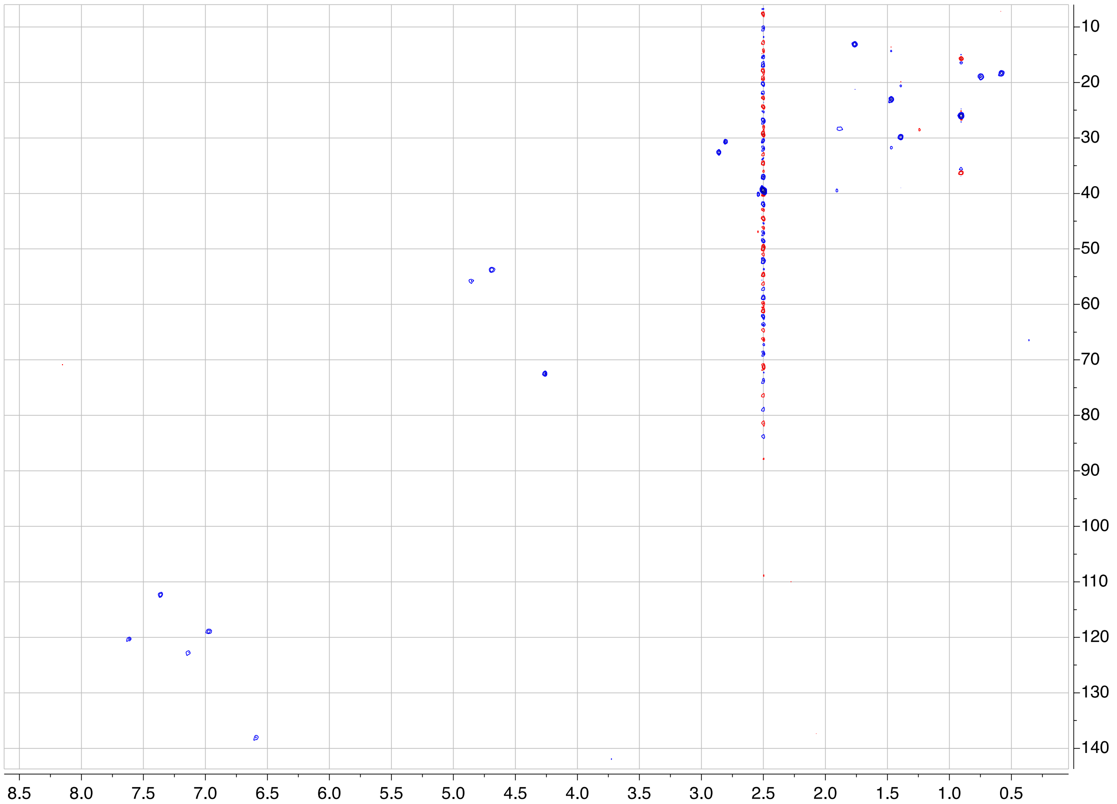

Figure S9. gHMBCAD Spectrum for milnamide F (2) in DMSO-d_6.

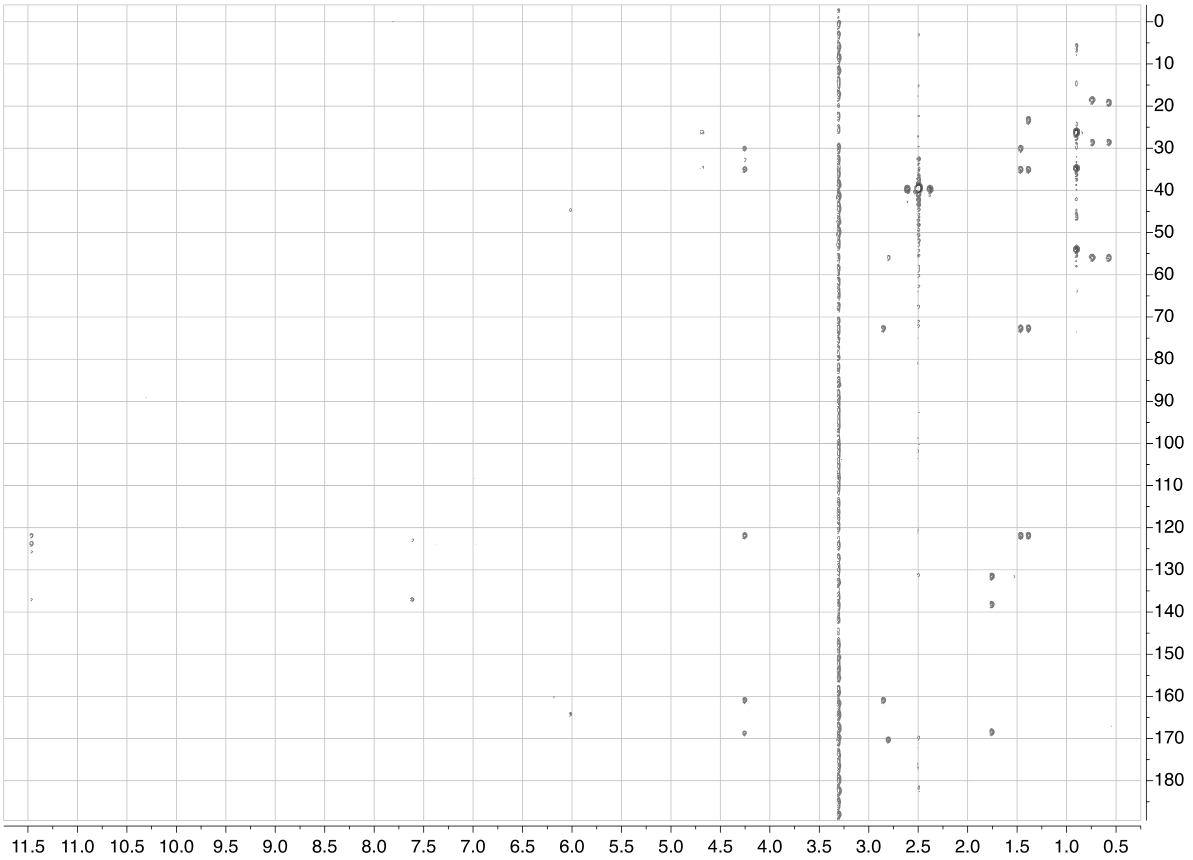

Figure S10. gCOSY Spectrum for milnamide F (**2**) in DMSO-d_6.

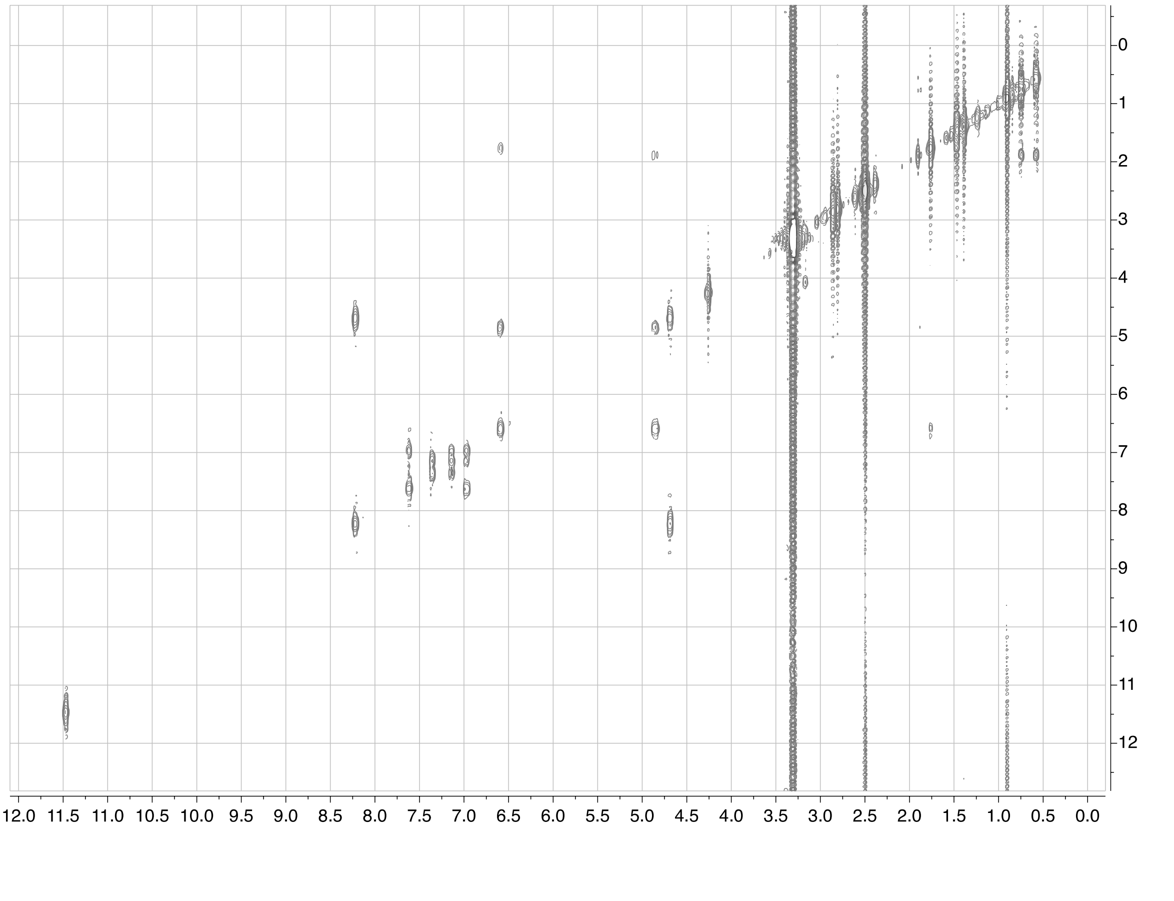

Figure S11. ROESY Spectrum for milnamide F (**2**) in DMSO-d_6.

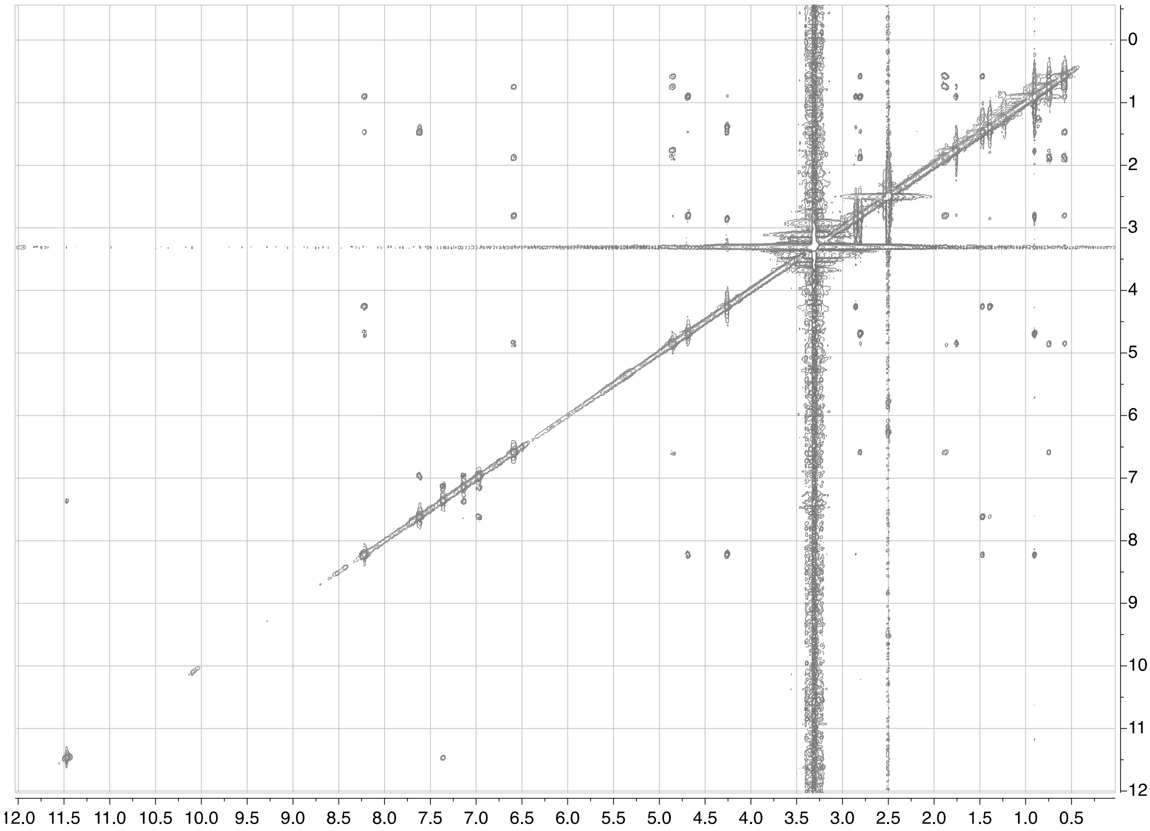

Figure S12. ^{1}H NMR Spectrum for milnamide G (**3**) in DMSO-d_6.

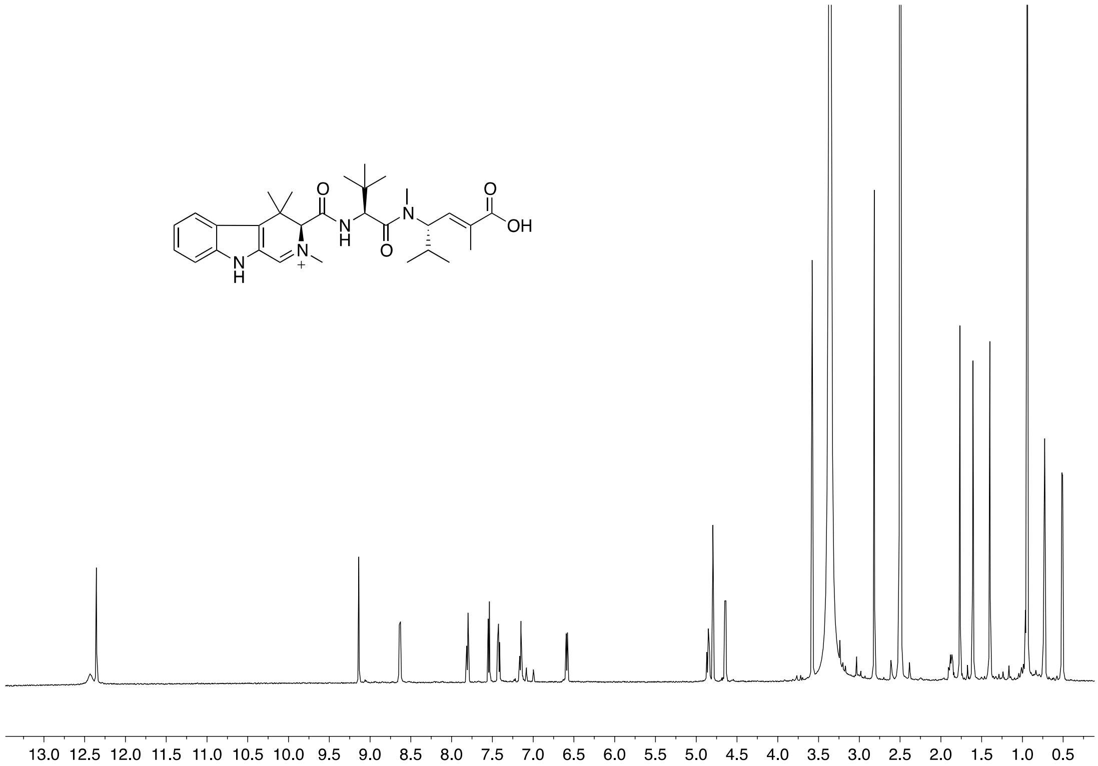

Figure S13. gHSQCAD Spectrum for milnamide G (**3**) in DMSO-d_6.

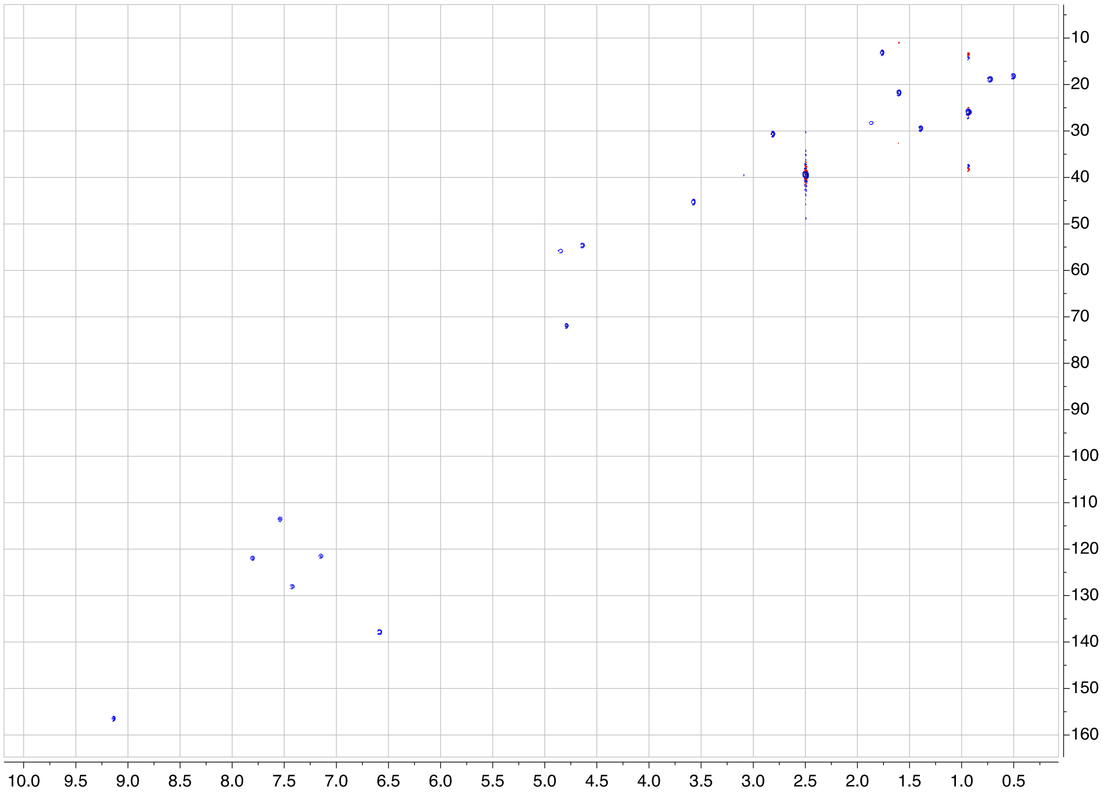

Figure S14. gHMBCAD Spectrum for milnamide G (**3**) in DMSO-d_6.

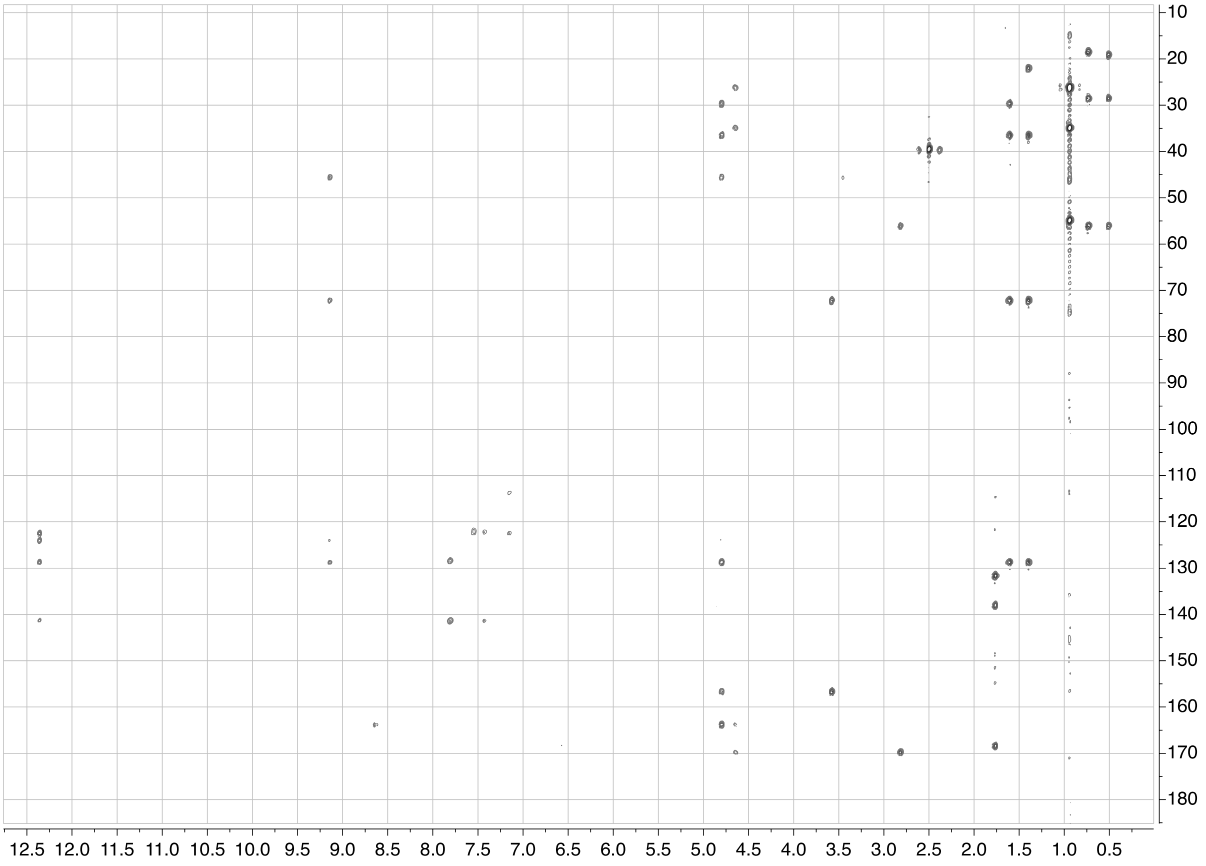

Figure S15. gCOSY Spectrum for milnamide G (**3**) in DMSO-d_6.

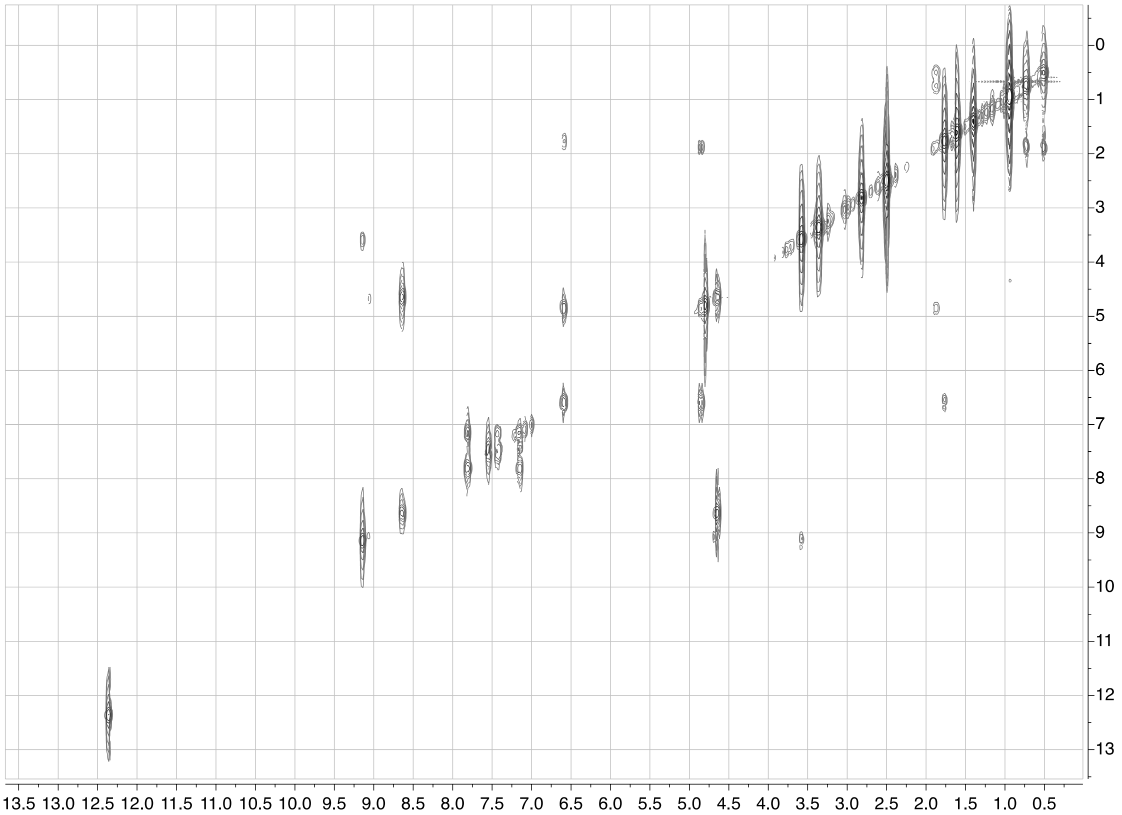

Figure S16. ROESY Spectrum for milnamide G (**3**) in DMSO-d_6.

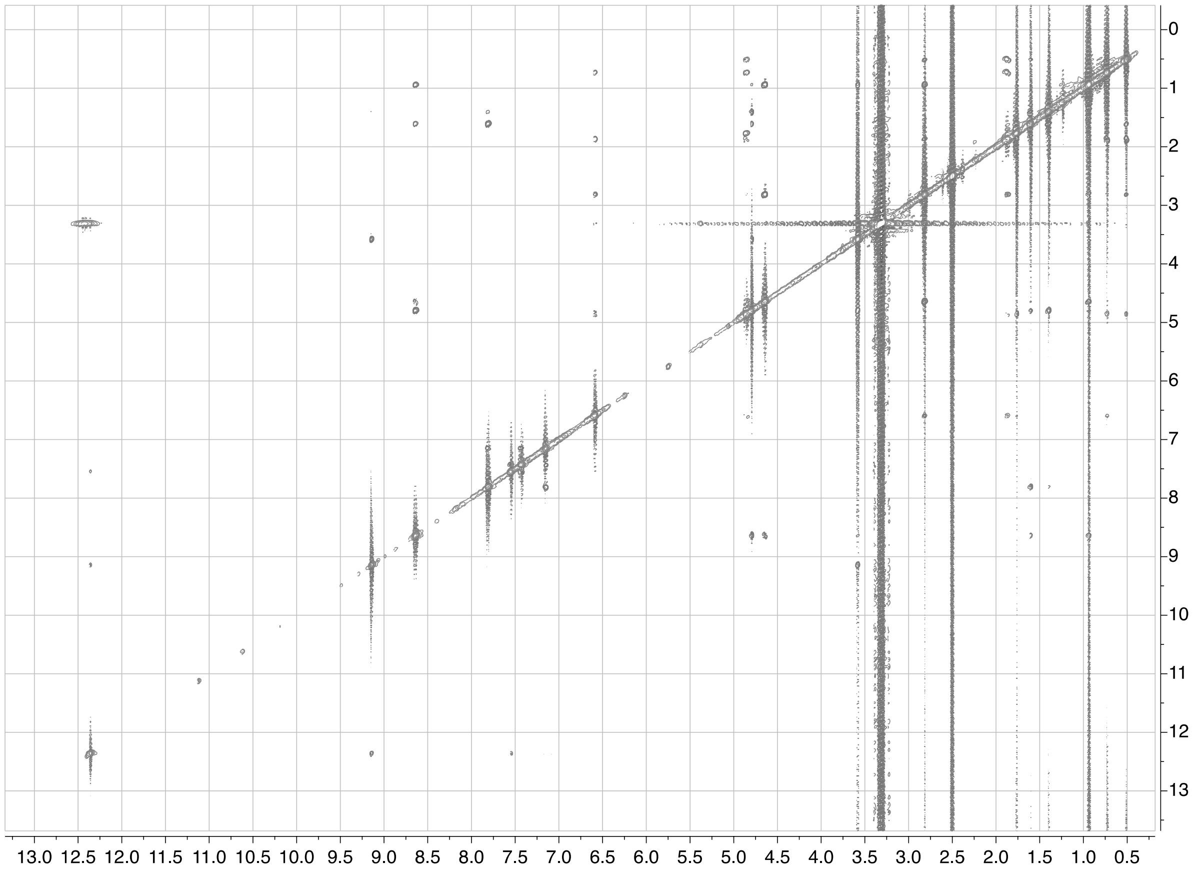

Figure S17. ^{1}H NMR Spectrum for hemiasterlin D (**4**) in DMSO-d_6.

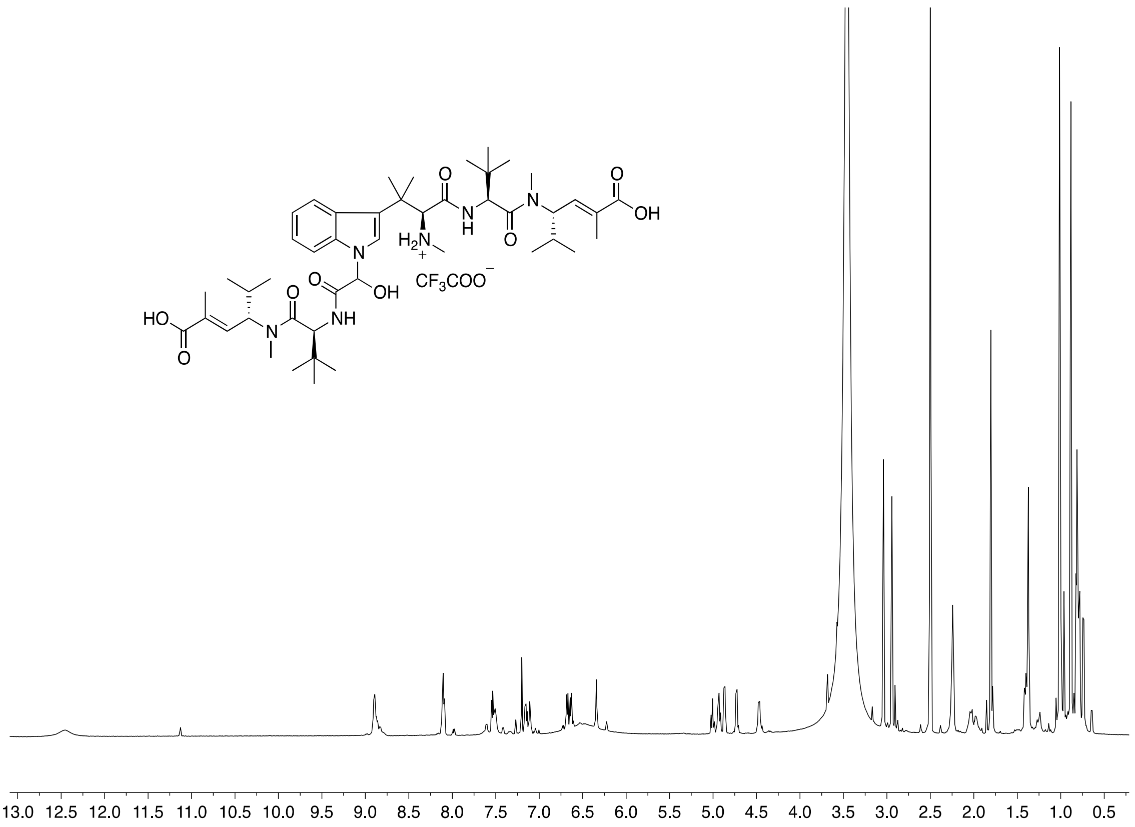

Figure S18. gHSQCAD Spectrum for hemiasterlin D (**4**) in DMSO-d_6.

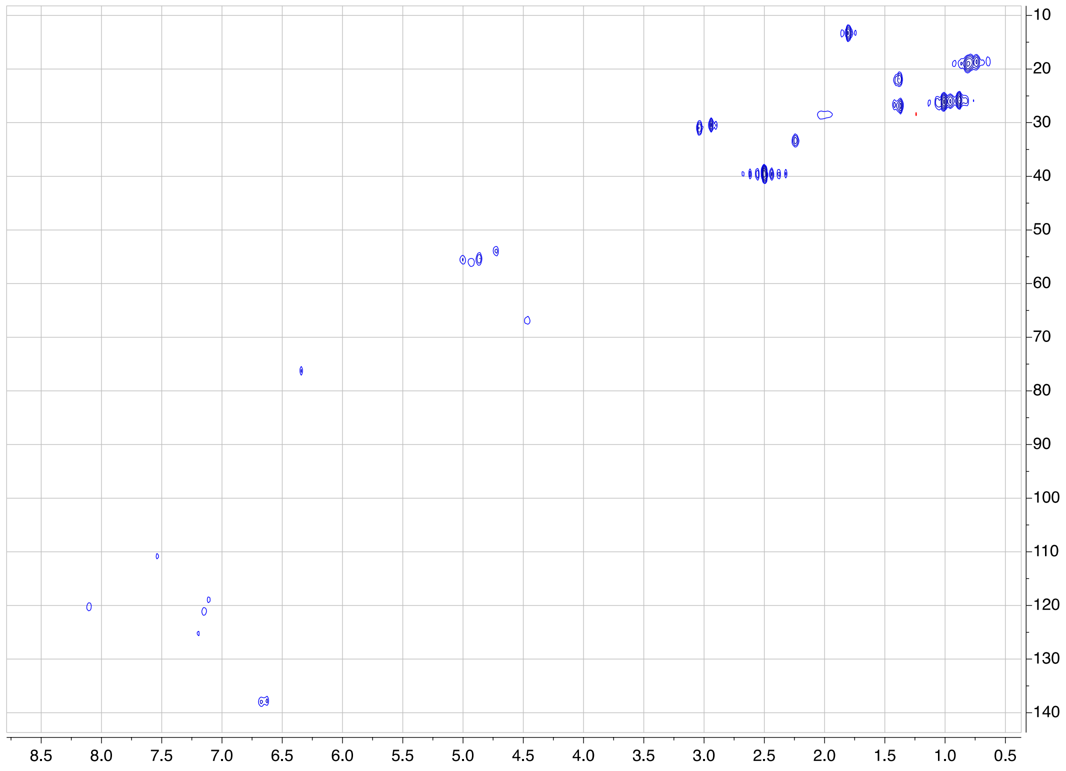

Figure S19. gHMBCAD Spectrum for hemiasterlin D (**4**) in DMSO-d_6.

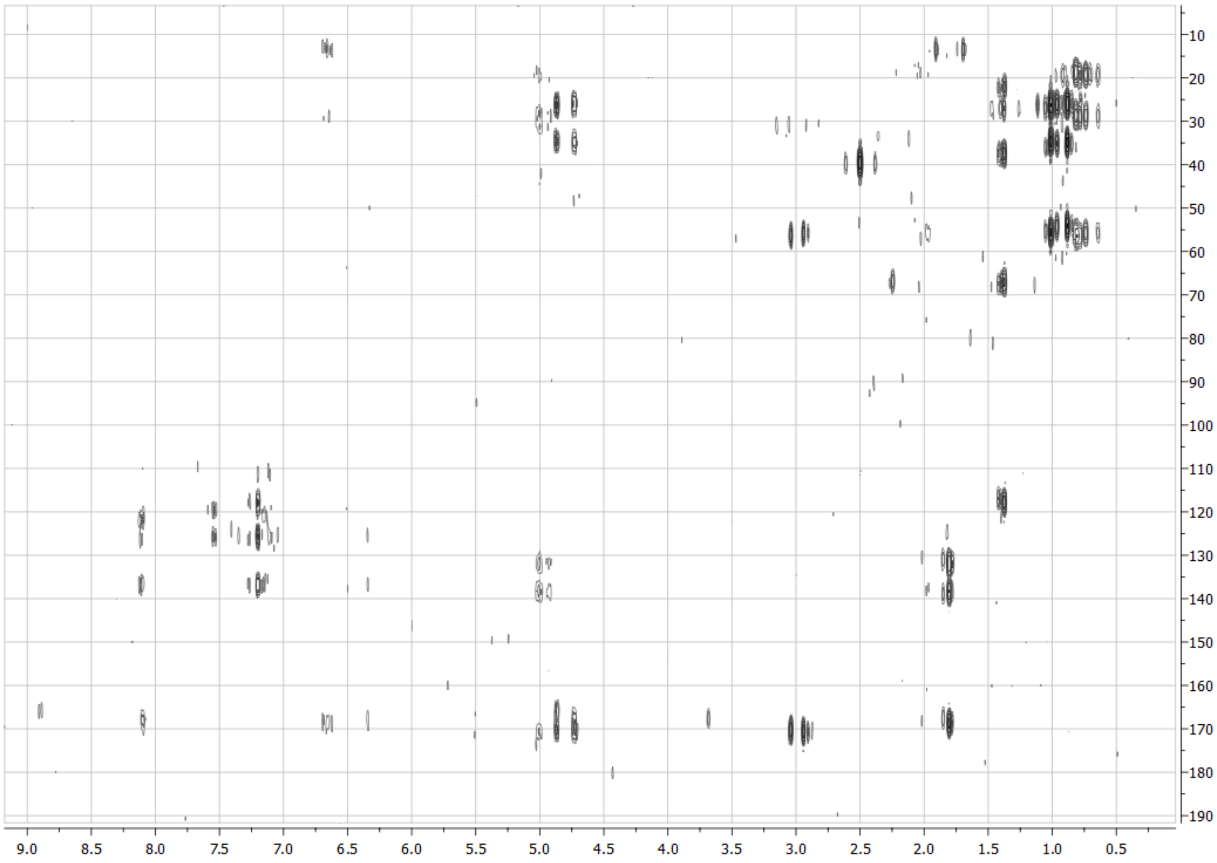

Figure S20. gCOSY Spectrum for hemiasterlin D (**4**) in DMSO-d_6.

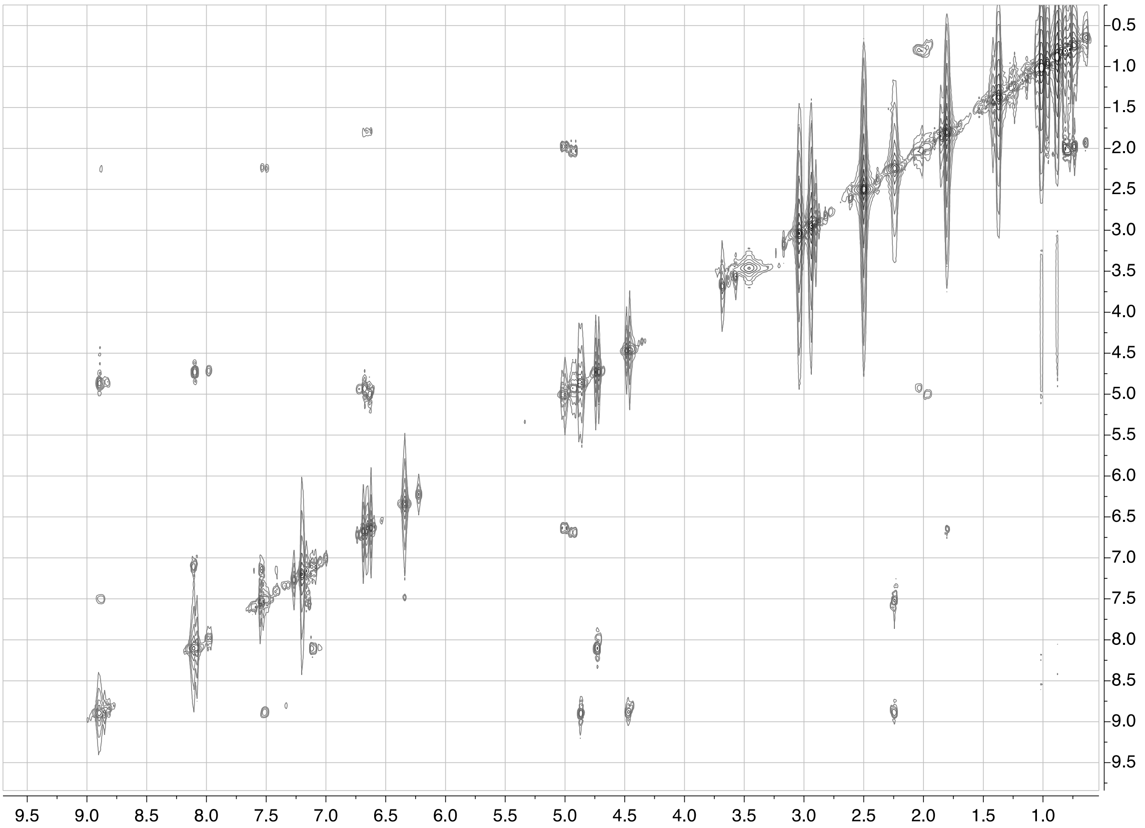

Figure S21. ROESY Spectrum for hemiasterlin D (**4**) in DMSO-d_6.

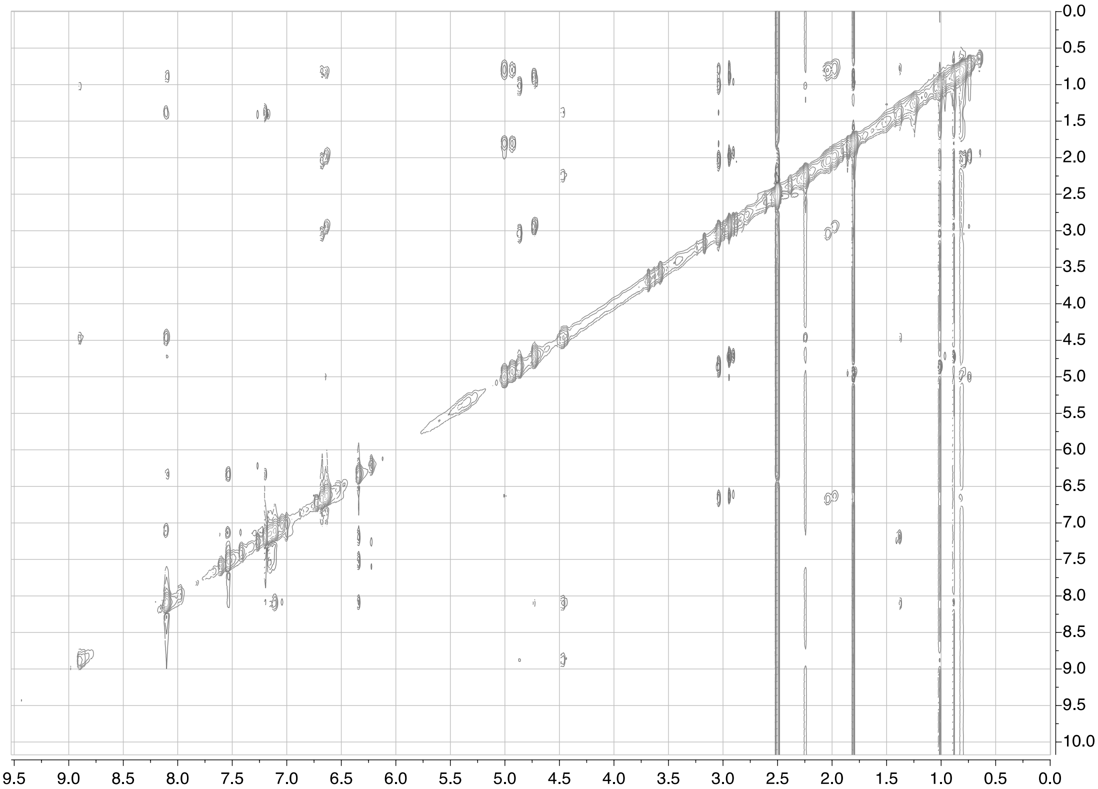

Figure S22. (+)-HRESIMS Spectrum for hemiasterlin D (**4**).

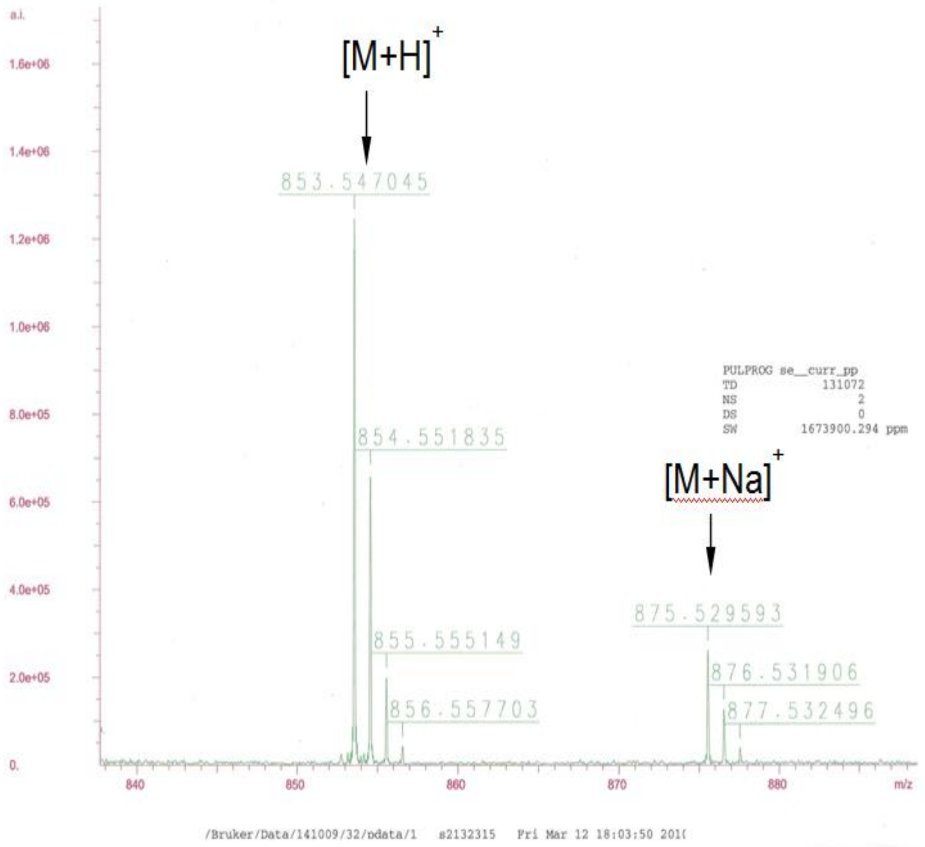

Figure S23. LC/MS Analysis of Marfey's Derivatives of compounds **1**–**4**.

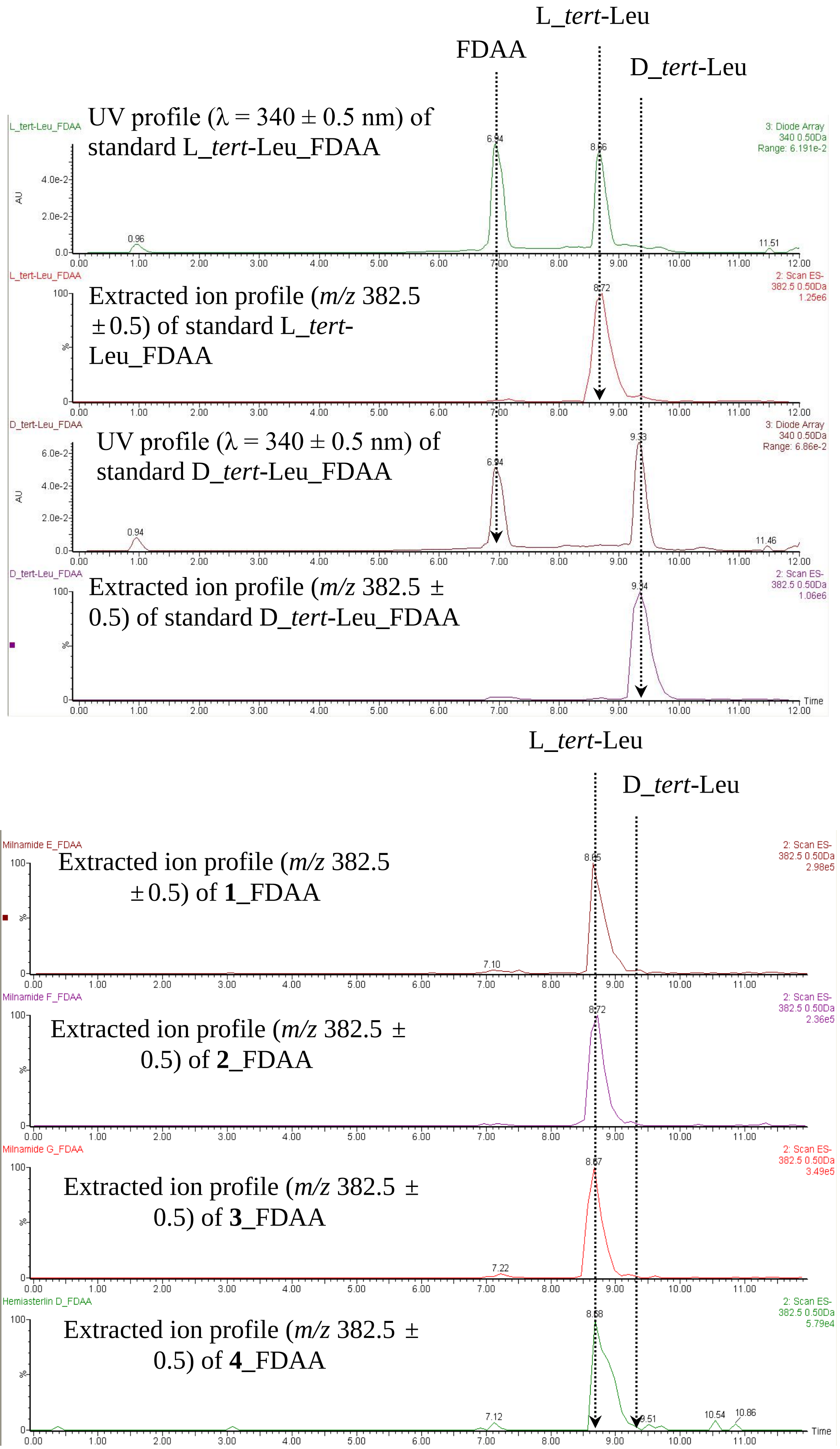

Figure S24. CD Spectra and Specific Rotations of Compounds **1**–**3** and **5**–**7**.

CD Spectra

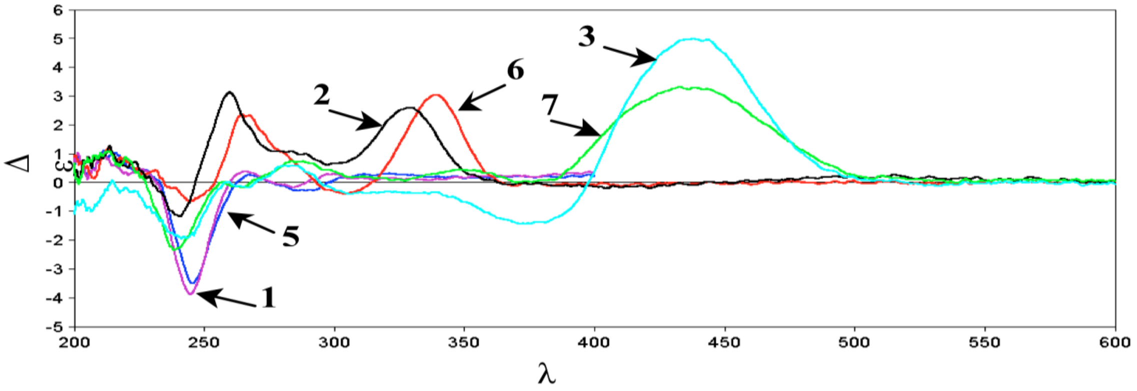

Specific Rotations

Compound	$[\alpha]^{25}_{D}$	$[\alpha]_{D}$ (Reference)
**1**	+11 (*c* 0.02, MeOH)	
**2**	+29 (*c* 0.05, MeOH)	
**3**	+134 (*c* 0.04, MeOH)	
**5**	+38 (*c* 0.02, MeOH)	+28.8 (*c* 0.50, CH_2Cl_2) [1]
**6**	+49 (*c* 0.05, MeOH)	+68 (*c* 0.06, MeOH) [2]
**7**	+180 (*c* 0.06, MeOH)	+156 (*c* 0.39, MeOH) [3]

Figure S25. Photographs of the sponges *Pipestela candelabra*; (**A**) Photograph of the sponge *Pipestela candelabra* collected at Wilson Reef, Coral Sea; (**B**) Photograph of the sponge *Pipestela candelabra* collected at Houghton Reef, Howick Group.

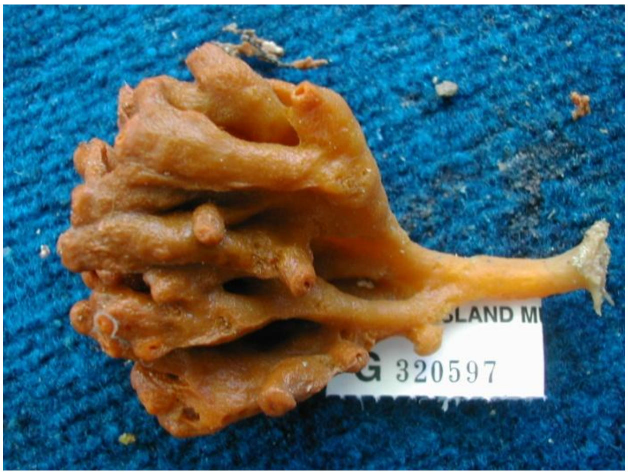

(**A**)

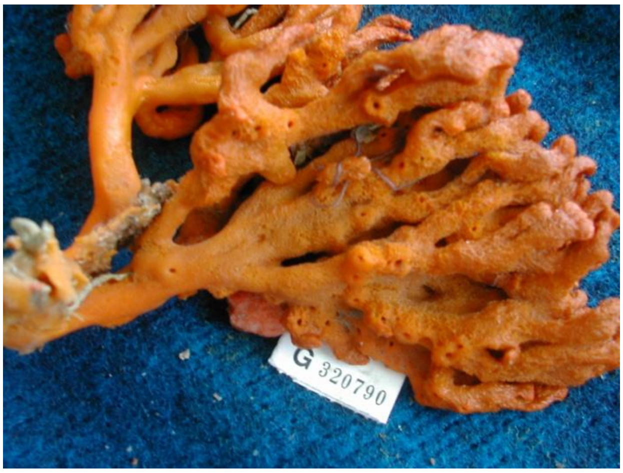

(**B**)

References

1. Crews, P.; Farias, J.J.; Emrich, R.; Keifer, P.A. Milnamide A, an unusual cytotoxic tripeptide from the marine sponge *Auletta* cf. *constricta*. *J. Org. Chem.* **1994**, *59*, 2932–2934.
2. Sonnenschein, R.N.; Farias, J.J.; Tenney, K.; Mooberry, S.L.; Lobkovsky, E.; Clardy, J.; Crews, P. A further study of the cytotoxic constituents of a milnamide-producing sponge. *Org. Lett.* **2004**, *6*, 779–782.
3. Chevallier, C.; Richardson, A.D.; Edler, M.C.; Hamel, E.; Harper, M.K.; Ireland, C.M. A new cytotoxic and tubulin-interactive milnamide derivative from a marine sponge *Cymbastela* sp. *Org. Lett.* **2003**, *5*, 3737–3739.

© 2014 by the authors; licensee MDPI, Basel, Switzerland. This article is an open access article distributed under the terms and conditions of the Creative Commons Attribution license (http://creativecommons.org/licenses/by/3.0/).
